# Identification of a dioxin-responsive oxylipin signature in roots of date palm: involvement of a 9-hydroperoxide fatty acid reductase, caleosin/peroxygenase PdPXG2

**DOI:** 10.1038/s41598-018-31342-4

**Published:** 2018-09-04

**Authors:** Abdulsamie Hanano, Mouhnad Shaban, Ibrahem Almousally, Denis J. Murphy

**Affiliations:** 1 Department of Molecular Biology and Biotechnology, Atomic Energy Commission of Syria (AECS), Damascus, Syrian Arab Republic; 2 Genomics and Computational Biology Group, University of South Wales, Wales, UK

## Abstract

Dioxins are highly hazardous pollutants that have well characterized impacts on both animal and human health. However, the biological effects of dioxins on plants have yet to be described in detail. Here we describe a dioxin-inducible caleosin/peroxygenase isoform, PdPXG2, that is mainly expressed in the apical zone of date palm roots and specifically reduces 9-hydroperoxide fatty acids. A characteristic spectrum of 18 dioxin-responsive oxylipin (DROXYL) congeners was also detected in date palm roots after exposure to dioxin. Of particular interest, six oxylipins, mostly hydroxy fatty acids, were exclusively formed in response to TCDD. The DROXYL signature was evaluated *in planta* and validated *in vitro* using a specific inhibitor of PdPXG2 in a root-protoplast system. Comparative analysis of root suberin showed that levels of certain monomers, especially the mono-epoxides and tri-hydroxides of C16:3 and C18:3, were significantly increased after exposure to TCDD. Specific inhibition of PdPXG2 activity revealed a positive linear relationship between deposition of suberin in roots and their permeability to TCDD. The results highlight the involvement of this peroxygenase in the plant response to dioxin and suggest the use of dioxin-responsive oxylipin signatures as biomarkers for plant exposure to this important class of xenobiotic contaminants.

## Introduction

Generically termed dioxins, the polychlorinated dibenzo-*p*-dioxins (PCDDs) and polychlorinated dibenzofurans (PCDFs), are collectively the most toxic group of Persistent Organic Pollutants (POPs) that have been described to date^1^. These halogenated contaminants are mainly released to the environment due to poorly managed incineration of industrial, municipal and domestic wastes^2^, and also during industrial processes involving chlorinated aromatic and aliphatic compounds, such as the chemical synthesis of pesticides and herbicides^3^. Structurally, dioxins consist of two aromatic rings linked via one (PCDFs) or two atoms of oxygen (PCDDs) and also contain one to eight chlorine atoms, which confers both high stability and extreme hydrophobicity. Depending on the number and position of chlorination (P = 1–8), the dioxin group comprises 75 PCDD and 135 PCDF congeners that vary greatly in terms of their toxicity to living organisms. Congeners with chlorine atoms positioned in 2, 3, 7 and 8 of the aromatic rings are the most toxic and 2,3,7,8-Tetrachlorodibenzo-*p*-dioxin (TCDD), with a toxic equivalency factor (TEF) of 1.0, is by far the most toxic of all the dioxins^4,5^.

Because they are so chemically stable, dioxins can persist in the environment and bioaccumulate in many different organisms in a given ecosystem, including bacteria, fungi, plants, insects, and larger animals including humans^6–9^. In mammals, dioxins mainly accumulate in fatty tissues due to their high lipophilicity. For example, dioxins are present at very high levels in liver adipose tissue and in milk lipid droplets (LDs)^10^ but are found in relatively low levels in brain tissue^7^. The affinity of dioxins towards lipids is also modulated by the lipid class. Hence, dioxins are accumulated to much higher levels in triacylglycerol assemblies (such as LDs) than in phospholipids (such as bilayer membranes)^11^. Dioxins therefore can seriously affect lipid metabolism in exposed mammals. For example, exposure to TCDD increases membrane lipid oxidation and phospholipase (PLA2) activity, which in turn increases levels of free arachidonic acid (AA), the metabolites of which can act as potent pro-inflammatory mediators^12–15^. Furthermore, TCDD can also modify AA metabolism downstream of PLA2 by inducing AA-metabolizing enzymes such as cytochrome P450, cyclooxygenases (COXs) and, probably, lipoxygenases (LOXs)^16–18^.

It is likely that plants are exposed to dioxins initially in contaminated environments and that animals become exposed subsequently, often via ingestion of plant tissues. Hence the bioaccumulation of these xenobiotics in plant tissues not only may seriously impact plant health, but also can contribute to their bio-transmission into the wider food chain. Although knowledge of plant-dioxin interactions lags far behind that for animals, attention has been recently paid to the biological and toxicological consequences of the exposure of plant to dioxins. Although they cannot be used for nutrition or as a source of energy, dioxins can be efficiently taken up and accumulated in plant tissues^19–21^. We have previously reported that Arabidopsis plants can take up dioxins, from artificially contaminated environments, and mainly accumulate them in leaves, seeds and roots. Furthermore, TCDD-induced toxicity was demonstrated through decreases in seed germination, plant fresh weight, and chlorophyll content, alongside an increase in the biomass of the lateral root system and enhanced levels of hydrogen peroxide H_2_O_2_ production, a massive stimulation of anti-oxidative enzyme activities, a delay in flowering and reduced yields of seeds that had low oil contents and low viability^22,23^. In terms of lipid metabolism, TCDD caused significant reductions in C18-unsaturated fatty acid levels in plant tissues that was accompanied by an induction in the expression of *9-LOX* and *13-LOX* genes and the formation of 9- and 13-HpODE as well as 9- or 13-HpOTrE, hydroperoxides derived from linoleic and linolenic acids, respectively.

It is probable that dioxins interact with plants at the subcellular level via their lipid components, the most notable of which are the cytosolic lipid droplets (LDs) that are ubiquitous in most living organisms^24^. For example, we have demonstrated that seed LDs extracted from date palm seeds are very effective sequestration agents for the dioxin, TCDD, and that exposure of date palm seedlings to TCDD resulted in a strong transcriptional induction of several members of the caleosin/peroxygenase gene family. We have shown that these caleosin/peroxygenases actively metabolize FA-hydroperoxides in plant cells and are localized both on the endoplasmic reticulum (ER) and on lipid droplets^25–28^. Plant and fungal caleosins/peroxygenases have distinctive features, including, a single highly conserved calcium binding, EF-hand motif plus an invariant heme-binding histidine residue in the region proximal to the N terminus. This is followed by a relatively hydrophobic, potentially membrane-spanning, region plus a proline rich domain in the centre of the protein. Finally, there is a region containing several predicted kinase sites proximal to the C terminus^25,29–31^.

Caleosins from both plants and fungi have lipid peroxygenase (PXG) activity that depends on the presence of calcium and a heme group coordinated by two invariant histidine residues^32,33^. The PXG catalyzes the transfer of an oxygen atom from a FA-hydroperoxide via an intra- or inter-molecular mechanism to form the corresponding FA-hydroxide^34^. Following emerging data on the involvement of PXG-derived hydroxides in the regulation of oxidative status in plants, a new concept for wider biological roles of PXG activity has been proposed^27^.

While extensive attention that has been paid to certain branches of plant oxylipin biosynthesis, such as those involving allene oxide synthase (AOS) and hydroperoxide lyase (HPL) that lead to jasmonates and aldehydes, respectively, relatively little work has been done on oxylipins that derive from the PXG pathway, whose physiological roles have been revealed only recently^27,33^. As shown in Figure S1, linoleic acid (C18:2)-derived oxylipins are formed under the action of PXG pathway where PXG can epoxidize mono- and/or poly-unsaturated fatty acids to produce their respective fatty acid epoxides, some of which have antifungal activities^35^. Subsequently, such FA-epoxides can be hydrolyzed to the corresponding FA-hydroxides by an epoxide hydrolase (EH)^36^. In addition to their antifungal properties, fatty acid di- and poly-hydroxides are required for formation of the plant cuticle, an extracellular lipidic network covering aerial parts of most plants^37^. Another catalytic feature of PXG is that this enzyme can reduce, via an intra- or inter-molecular mechanism, 9- and 13-fatty acid hydroperoxides to their corresponding hydroxide-epoxide- or hydroxide-fatty acids, respectively. These FA-hydroxides, are known to act as signal molecules and play pivotal roles in controlling the oxidative status of plant cells^27^. In addition to its multiple enzymatic activities, PXG has been shown to have a role in the biogenesis and stabilisation of cytosolic lipid droplets (LDs) where it probably acts in conjunction with other major LD-associated proteins such as oleosins and steroleosins to prevent LD coalescence/aggregation^38^. A similar range of biological functions for PXGs have recently emerged in fungi where a newly characterized PXG has been implicated in a new oxylipin biosynthetic pathway and to be involved in the overall development, conidia formation and aflatoxin production in *Aspergillus flavus*^39^.

Oxylipin biosynthesis has mainly been described in leaves^40^, but there is increasing evidence of the presence of the complete biosynthetic pathway machinery in the roots^41^. In the case of root systems, some the most relevant abiotic stresses that might involve oxylipins include salinity, dehydration, and exposure to heavy metals and other toxic compounds. It is possible that PXGs are involved in plant responses to dioxins at several different levels. For example, PXGs might help stimulate LD accumulation in order to assist toxin sequestration on the droplets. Alternatively, or perhaps additionally, PXGs may act as part of an oxylipin signalling pathway that is involved in the overall stress response to the toxin^42^. Two PXG isoforms were recently characterised in roots of date palm, *Phoenix dactylifera* L., seedlings with respect to their tissue expression, subcellular localization and lipid metabolism. Both caleosins were localized into LDs and microsomal fractions and had peroxygenase activities that were strongly induced by TCDD and they were named as PdPXG2 and PdPXG4.

In this study, we report the biochemical and catalytic characterization of isoform PdPXG2, which was actively expressed in healthy roots of date palm seedlings and highly induced in response to TCDD exposure. This PXG is shown to be a novel 9-hydroperoxide fatty acids reductase involved in the response of roots to TCDD-exposure via its enzymatic activity. We also establish a specific and reproducible “signature” of oxylipins formed due to the toxin-induced activity of PdPXG2. The use of PdPXG2-derivatives oxylipins as “lipid biomarkers” indicating the exposure of plant root to dioxin was also validated in root protoplasts. Finally, we discuss the implications of the TCDD-responsive oxylipin induction on the deposition and composition of root suberin monomers, and hence their permeability against exogenous TCDD.

## Results

### PdPXG2 is structurally a typical plant peroxygenase

**Figure 1:**
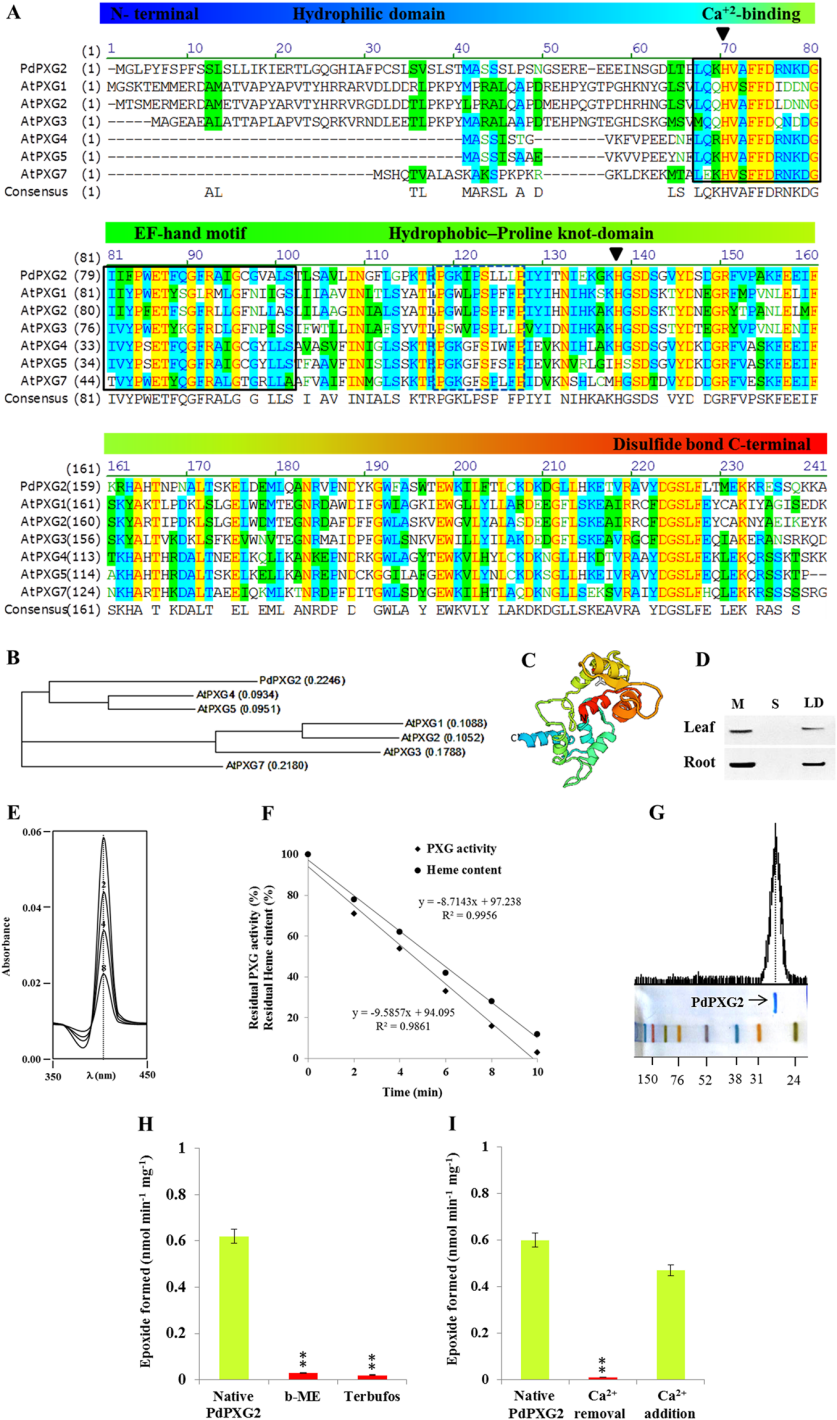
Sequence analysis and biochemcal properities of PdPXG2. (**A**) Multiple alignment of the PdPXG2 sequence with *A*. *thaliana* AtPXG1 (At4g26740), AtPXG2 (At5g55240), AtPXG3 (At2g33380), AtPXG4 (At1g70670), AtPXG5 (At1g70680) and AtPXG7 (At1g23240). The boxed areas correspond to the Ca^2+^ binding and the proline knot domains. Triangles indicate positions of Histidines H_68_ and H_136_ responsible to heme binding. (**B**) Phylogenetic tree of PdPXG2 with its orthologs in *A*. *thaliana*. (**C**) Predicted 3D protein structure of PdPXG2. The virtual image was generated online using http://www.sbg.bio.ic.ac.uk/phyre2. Image was rainbow colored from N to C terminus. (**D**) Immunoblotting of PdPXG2 protein by a polyclonal antibody prepared from the complete sequence of AtCLO1 caleosin from *Arabidopsis thaliana*, diluted 1:500 in TBS buffer (pH 7.4). (**E**) Sequential scanning of the heme-spectrum of PdPXG2 obtained by addition of cumene hydroperoxide at the indicated times. (**F**) Correlation between the epoxidation activity of purified PdPXG2 and disappearance of the heme content. (**G**) PdPXG2 was radio-phosphorylated by casein kinase and [^35^S]ATP. (**H**). Inhibition of native PdPXG2 was by treatment with β-mercaptoethanol (β-MPE) (1 mM) or terbufos (3 mM). (**I**) Activities for native purified PdPXG2 and Ca^2+^-deionized PdPXG2 after extensive dialysis and for PdPXG2 after dialysis followed by addition of 1 mM CaCl_2_ to the medium. All measurements were in triplicate. Values are the means ± S.D. (*n* = 3). Asterisks indicate significant differences in epoxidation activities between treated and native PdPXG2 (**P < 0.01).

We previously expressed His-tagged PdPXG2 in yeast cells where the enzymatic fraction was solubilized, using 0.2% emulphogene, purified by Ni^2+^-affinity chromatography and analyzed by SDS-PAGE to yield a major single protein band at about 27 kDa^43^. Aligned to protein sequences of caleosins from *Arabidopsis thaliana*, PdPXG2 was most similar to AtPXG5 and AtPXG4 with 59.9 and 53.9% identities respectively and least similar to AtPXG1 and AtPXG2 with 33.1 and 34.2% identities respectively (Fig. 1A). As expected for a caleosin, PdPXG2 contains an N-terminal calcium-binding EF-hand and a proline-rich domain (PXXXPSPXXP) in the central hydrophobic region that has been postulated to be essential for caleosin anchoring to LDs and bilayer membranes^44^. In addition, two essential histidine residues for plant peroxygenase activity are present (H_68_ and H_136_), supporting that this caleosin might also act as a peroxygenase. Phylogenetic analysis confirmed that PdPXG2 clusters in the same branches as AtPXG5 and AtPXG4 (Fig. 1B) and the predicted 3D-structure of the PdPXG2 protein shows a domain configuration which significantly matches that of other plant caleosins (Fig. 1C). PdPXG2 is most abundant in microsomes (M), less so in LDs, and was absent from supernatant (S) fractions isolated from roots of date palm seedlings compared with the respective fractions isolated from leaves (Fig. 1D). The peroxygenase activity has been assigned to a heme prosthetic group presumably bound to histidine residues of the active site of the caleosin. The preservation of these both histidines (H_68_ and H_136_) in the primary structure of PdPXG2 suggested that this protein might also contain a heme group. This was confirmed by analysis of light absorption spectra that contained a 407 nm peak characteristic of the Soret band of hemoproteins (Fig. 1E). Moreover, the abstraction of the heme coordination complex by addition of 1 mM cumene hydroperoxide resulted in a gradual decrease in the absorbance at 407 nm that was well correlated with subsequent enzyme inactivation (Fig. 1F). In addition, PdPXG2 was radio-phosphorylated by casein kinase and [^35^S]ATP, although this did not result in any changes in its enzymatic activity (Fig. 1G). The oxidative function of PdPXG2 was abolished by addition of either 1 mM β-mercaptoethanol or 3 mM terbufos, which are well known competitive and suicide inhibitors, respectively, of plant PXGs (Fig. 1H). Finally, the removal of Ca^+2^ by dialysis of PdPXG2 against EDTA completely abolished its co-oxidative properties, which were restored (by up to 78.1%) by re-adding 1 mM CaCl_2_ (Fig. 1I). Altogether, these results indicate that PdPXG2 is indeed a typical plant peroxygenase.

### PdPXG2 has a novel form of peroxygenase activity

**Figure 2:**
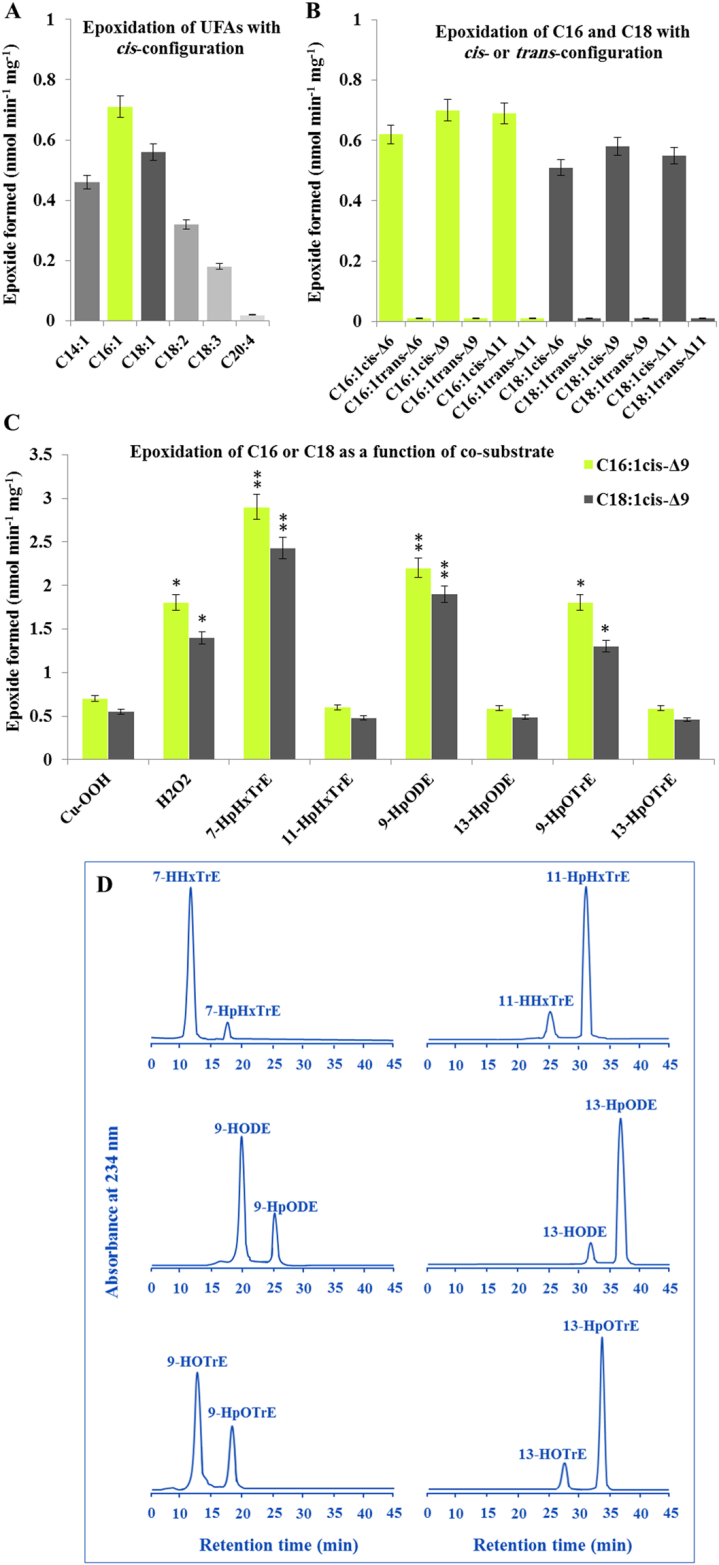
Catalytic properities of PdPXG2. (**A**) Fatty acid epoxidation activity of PdPXG2 as a function of carbon chain length and unsaturation degree of fatty acids. (**B**) Stereoselectivity of PdPXG2 towards the *cis*-double bond configuration in C16:1 or C18:1. PdPXG2 exhibited a strong stereoselectivity since no epoxidation was detected with UFAs having the double bond in *trans*-configuration regardless of the position (6, 9 or 11) of the *cis*-double bond in the carbon chain. (**C**) Epoxidation of [^14^C]-labelled palmitic acid (C16:1) and oleic acid (C18:1) (*Cis*-Δ9 for both) by recombinant PdPXG2 in the presence of various hydroperoxides; i.e. H_2_O_2_, 7-HpHxTrE, 11-HpHxTrE, 9-HpODE, 13-HpODE, 9-HpOTrE and 13-HpOTrE compared with cumene hydroperoxide (Cu-OOH). (**D**) The reductase activity of purified recombinant PdPXG2 against fatty acid hydroperoxides, 7-HpHxTrE, 11-HpHxTrE, 9-HpODE, 13-HpODE, 9-HpOTrE and 13-HpOTrE using a HPLC-UV-detector system. All measurements were in triplicate. Values are the means ± S.D. (*n* = 3). *P < 0.05; **P < 0.01.

The activity of PdPXG2 to epoxidize various unsaturated fatty acids (UFAs) (Table S2) was determined. First, the epoxidation of double bonds was evaluated in UFAs, which had similar double bond numbers but varied in chain length and degree of unsaturation. Unlike most of the characterized plant PXGs, PdPXG2 preferentially epoxidized [^14^C]-labelled mono-UFAs C16:1, followed by C18:1 (>160 nmol of epoxide was formed min^−1^ mg^−1^ of protein). Shortening or extending the carbon chain with an increasing degree of unsaturation decreased the epoxidation of a given fatty acid (Fig. 2A). Moreover, moving the single *cis*-double bond of C16:1 or C18:1 to positions 6, 9 or 11 did not significantly affect epoxidation rates (Fig. 2B). This epoxidase also exhibited strong stereoselectivity with no epoxides detected from UFAs having the double bond in *trans*-configuration regardless the position of double bond in the carbon chain (Fig. 2B). It should be noted here that the methyl ester of palmitoleic acid was also an excellent substrate of the epoxidase (data not shown) indicating that a free carboxylic group is not necessary for the positioning of the substrate into the active site of PdPXG2. These data suggest that this PdPXG prefers the epoxidation of a double bond in *cis*-configuration with a carbon chain length of C16.

The epoxidase activity of plant PXG is normally dependent on the chemical nature of the co-substrate from which the PXG removes the molecular oxygen. To determine the best co-substrate that insures the optimal activity for PdPXG2, [^14^C]-labelled palmitic acid (C16:1) and oleic acid (C18:1) were individually incubated with recombinant PdPXG2 in the presence of various oxygen-donor-hydroperoxides; i.e. H_2_O_2_, 7-HpHxTrE, 11-HpHxTrE, 9-HpODE, 13-HpODE, 9-HpOTrE and 13-HpOTrE compared with the routinely used cumene hydroperoxide (Cu-OOH). Figure 2C shows that the most efficient co-substrates for PdPXG2 were the FAs-OOH derived from the 9-lipoxygenase pathways of C16:3, C18:2 and C18:3; i.e. 7-HpHxTrE, 9-HpODE and 9-HpOTrE, respectively, where the epoxidase activities were up to 2.9 nmol of C16-epoxide formed per min per mg of enzyme. Hydrogen peroxide, H_2_O_2_, was also a better co-substrate than Cu-OOH. Unexpectedly, the hydroperoxides derived from the 13-lipoxygenase pathways of the same FAs, i.e. 11-HpHxTrE, 13-HpODE and 13-HpOTrE were relatively poor co-substrates for PdPXG2, with activities not exceeding 0.6 nmol of C16-epoxide formed per min per mg of enzyme (Fig. 2C). These data indicate that PdPXG2 preferentially metabolizes 9-lipoxygenase-derived FA-hydroperoxides.

The reductase activity of purified recombinant PdPXG2 with fatty acid hydroperoxide substrates was determined using an HPLC-UV-detector system. Figure 2D shows that PdPXG2 reduced 7-HpHxTrE, 9-HpODE and to a lesser extent 9-HpOTrE, with only about 12%, 28% and 36% of the respective hydroperoxides remaining intact. But PdPXG2 showed a low reductase activity with11-HpHxTrE, 13-HpODE and 13-HpOTrE, with the highest activity detected for 11-HpHxTrE not exceeding 16% (Fig. 2D). The resulting FA-OHs were identified by co-elution with respective standards. No metabolites were detected from incubations of FA-OOHs with heat-inactivated PdPXG2 (data not shown). These measurements show that PdPXG2 most actively reduces FA-OOHs that result from 9-lipoxygenase pathways.

### PdPXG2 is mainly expressed in the apical zone of young roots

**Figure 3:**
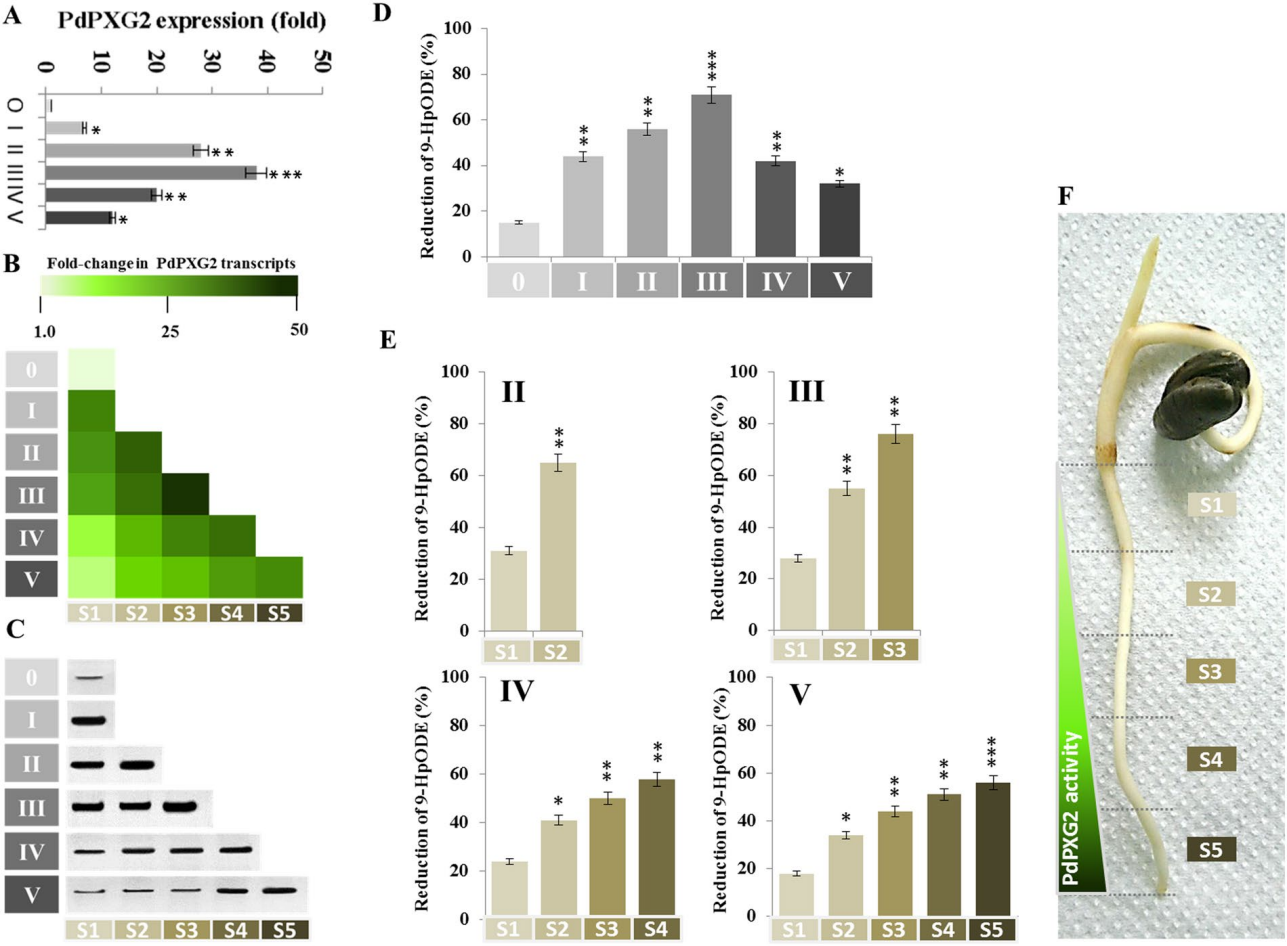
Expression and activity of PdPXG2 as a function of root development. (**A**) Quantification of *PdPXG2* transcripts in whole roots of date palm seedlings at different stages of development (from I to V) compared with non-germinated seeds (stage 0). For each stage, the transcript level was evaluated by qRT-PCR. (**B**) Spatiotemporal variations of *PdPXG2* gene expression ordered in the sections taken from the top to the root apical zones. (**C**) Immunoblotting of PdPXG2 protein was performed in the same samples used in B using a polyclonal antibody prepared from the complete sequence of AtCLO1 caleosin from *Arabidopsis thaliana*, diluted 1:500 in. (**D**) The enzymatic activity, measured as a 9-HpODE-reductase, followed the accumulation of PdPXG2 proteins in the extracts of whole roots at the indicated stages of development (I to V). (**E**) Spatiotemporal variations of 9-HpODE-reductase activity as a function of localization from the top to the root apical at stages (II, III, IV and V). (**F**) Image of a date palm seedling at stage V. The triangle shows the spatiotemporal variations of PdPXG2 activity along roots from the apical zone (dark-green) to the top (light-green). All measurements were in triplicate. Values are the means ± S.D. (*n* = 3). *P < 0.05; **P < 0.01; ***P < 0.001.

We recently reported that PdPXG2 was more highly expressed in roots than in plumules of date palm seedlings and suggested that PdPXG2 might function in a tissue-specific manner^43^. To verify this, levels of transcripts, proteins and enzymatic activity of PdPXG2 were analysed as a function of the root developmental stage and in different sections ordered from top to the root tip. Compared with non-germinated seeds (stage 0), transcripts of *PdPXG2* accumulated significantly in roots at stage I (7-fold), increased in stage II (28-fold) and peaked in stage III (39-fold) (Fig. 3A, top). Progressively the *PdPXG2* gene transcripts decreased in stages IV and V (20 and 12-fold, respectively). Spatially, the amount of *PdPXG2* transcripts were negatively ordered in sections taken from the top to the root apex, e.g., *PdPXG2* transcripts were about 38-fold less abundant in S1 compared with S5 in stage III (Fig. 3B). In parallel, while PdPXG2 proteins were detectable in the dormant seeds, they were much more abundant in the apical zone of roots at stage I and II (Fig. 3C) and reached maximal levels in the S3 of stage III. The enzymatic activity, measured as 9-HpODE-reductase, closely followed the accumulation pattern of PdPXG2 proteins with the lowest activity at stage 0 and the maximal at stage III (about 71% of 9-HpODE reduction) (Fig. 3D). The 9-HpODE-reductase activity was highest in sections from root apical zones for all stages (Fig. 3E, II, III, IV and V), which indicates that the expression and activity of PdPXG2 is more active in young roots and is particularly concentrated in the apical zone (Fig. 3F).

### PdPXG2 is induced by TCDD

**Figure 4:**
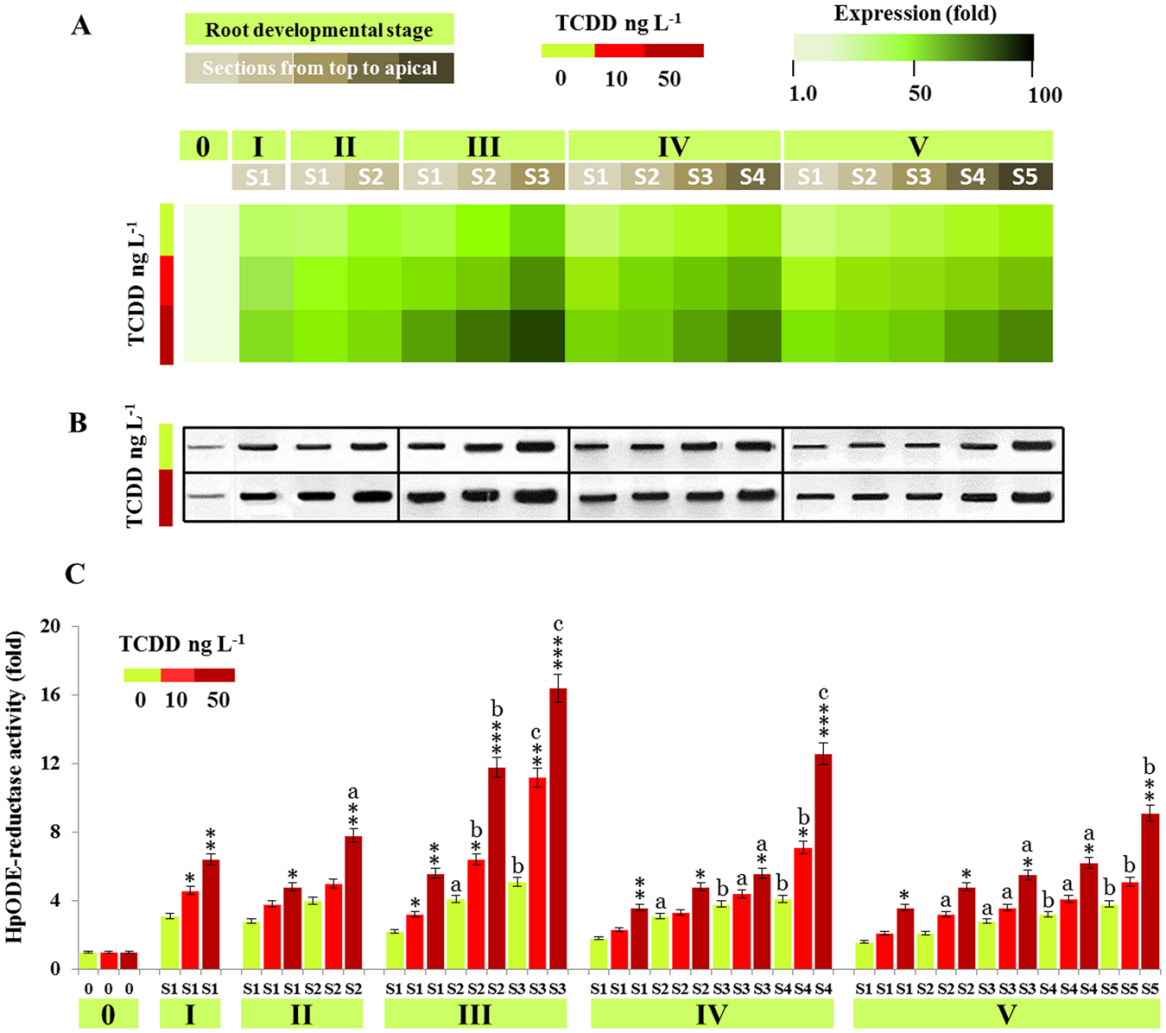
Expression and activity of PdPXG2 in the root of date palm seedling after exposure to TCDD. (**A**) Relative expression of *PdPXG2* in different sections (S1–S5) of roots at various developmental stages (I to V) after exposure to TCDD (0, 10 and 50 ng.L^−1^, light-green, light-red and dark-red, respectively). For each section, the transcript level was evaluated by qRT-PCR. Three measurements were taken in three cDNAs prepared from three individual plants for each treatment. The colour scale (white-green-black) indicates relative changes of transcript abundance of 1, 50 and 100 fold, respectively. The expression level in control samples was defined as 1, and corresponding abundance changes under 10 and 50 ng.L^−1^ TCDD were calculated directly using the Applied Biosystems qPCR system software. (**B**) Immunoblotting of PdPXG2 protein in the same samples of control and those treated with 50 ng.L^−1^ using a polyclonal antibody of AtCLO1 caleosin. (**C**) Spatiotemporal evaluation of 9-HpODE reductase activity as a function of section localization from the top to the root apical at stages (II, III, IV and V) after exposure to 10 and 50 ng.L^−1^ (light-red and dark -red, respectively) compared with controls (light-green). Activities were measured in triplicate. Values are the means ± S.D. (*n* = 3). Asterisks indicate significant differences in the tissue reductase-activity between TCDD-treatments and controls (*P < 0.05; **P < 0.01; ***P < 0.001). Lower cases indicate significant differences in the sectional reductase-activity between TCDD-treatments and controls (^a^*P* < 0.05; ^b^*P* < 0.01; ^c^*P* < 0.001).

We previously showed that accumulation of *PdPXG2* transcripts and HpODE-reductase activity were greatly enhanced in date palm seedlings grown in the presence of the dioxin, TCDD^43^. To study this further, we followed transcript and protein levels plus the enzymatic activity of PdPXG2 in different sections (S1–S5) at different stages of root development after exposure to TCDD. The heat map presented in Figure 4A shows a significant increase in levels of *PdPXG2* transcripts as a function of the TCDD-treatment, with the highest accumulation at stage III. Compared to non-treated roots, *PdPXG2* transcripts increased about 1.6 and 2.5-fold in roots exposed to 10 and 50 ng L^−1^ TCDD, respectively. Similarly to controls, TCDD-exposed roots accumulated more transcripts in the apical tip region. The increase in *PdPXG2* transcripts was correlated with accumulation of PXG proteins in the respective tissues (Fig. 4B). PXG activity in reducing 9-HpODE was stimulated in the same manner and increased in stage III, where this activity, normalized to control, was doubled and tripled in section S3 of the roots exposed to TCDD with 10 or 50 ng L^−1^, respectively (Fig. 4C). These data show that the PdPXG2 is induced in roots of date palm after exposure to dioxin and is most pronounced in the apical zone.

### PdPXG2-derived oxylipins are dominant in date palm exposed to TCDD

**Figure 5:**
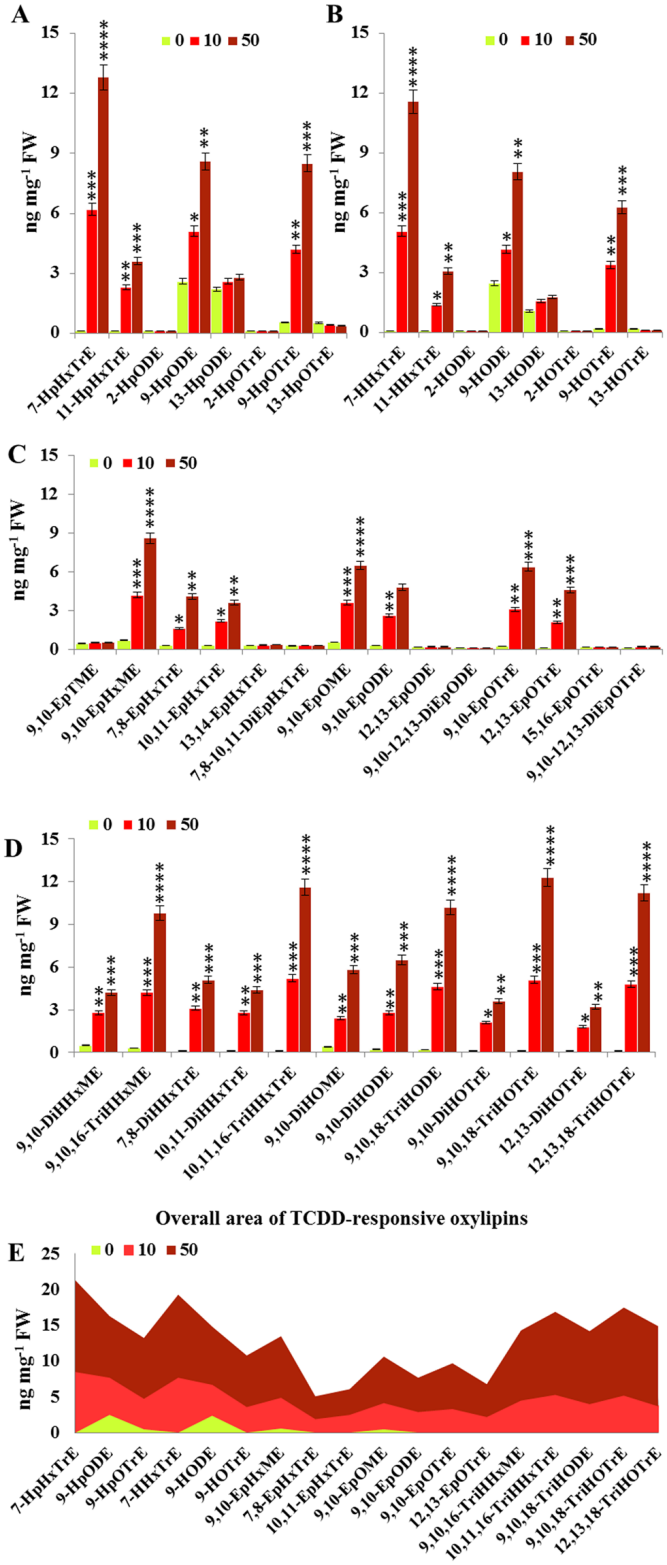
Quantification of PdPXG2-derivatives oxylipins in the date palm root after exposure to TCDD. (**A**) Amounts of fatty acid hydroperoxides (-OOH), expressed as ng mg^−1^ FW (fresh weight), resulting from oxygenation of C16:3, C18:2 and C18:3 under the action of 9-LOX, 13-LOX or α-DIOX in roots of date palm after exposure to TCDD at 10 ng.L^−1^ (light-red columns) or 50 ng.L^−1^ (dark -red columns) compared with control (light-green columns). (**B**) Amounts of fatty acid hydroxides (-OH) resulting from reduction of the indicated hydroperoxides in root tissues after exposure to TCDD compared with controls. (**C**) Amounts of mono- and di-epoxy fatty acids formed from C14:1, C16:1, C16:3, C18:1, C18:2 and C18:3 in root tissues in response to TCDD-exposure. (**D**) Amounts of di- and tri-hydroxy of C16:1, C16:3, C18:1, C18:2 and C18:3 in the same samples. (**E**) Overall evaluation of the quantitative areas covered by TCDD-induced oxylipin congeners after administration of a high (dark -red) or a low dose (light-red) of TCDD. All measurements were done in triplicate. Values are the means ± S.D. (*n* = 3). *P < 0.05; **P < 0.01; ***P < 0.001; ****P < 0.0001.

The activation of PdPXG2 in date palm roots after exposure to TCDD raises the question of the nature and possible roles of oxylipins formed by this peroxygenase. We therefore performed quantitative and qualitative analyses of oxylipins in control and TCDD-exposed roots. Compared to controls, TCDD-exposed roots accumulated significant amounts of fatty acid hydroperoxides, notably 7-HpHxTrE, 9-HpODE and 9-HpOTrE resulting from the oxygenation of C16:3, C18:2 and C18:3, respectively, under the action of 9-lipoxygenases (Fig. 5A). A considerable proportion of these hydroperoxides was reduced to the corresponding hydroxides, 7-HHxTrE, 9-HODE and 9-HOTrE, which were increased in TCDD-exposed roots to about 11.6, 8.1 and 6.3 ng mg^−1^ FW, respectively (Fig. 5B). Smaller amounts of 11-HpHxTrE, 13-HpODE and 13-HpOTrE and their respective hydroxides were also found while 2-HpOTrE and 2-HpODE and their corresponding hydroxides were undetectable in TCDD-exposed roots. Furthermore, only the mono-epoxide derivatives of C16:1, C16:3, C18:1, C18:2 and C18:3 were significantly increased in TCDD-treated roots (Fig. 5C). There was a particularly strong increase (13–21 fold) in 9,10-epoxides in roots exposed to 50 ng L^−1^ compared with controls. Substantial accumulation of some tri-hydroxides fatty acids, e.g., 9,10,16-TriHHxME, 10,11,16-TriHHxTrE, 9,10,18-TriHODE and 12,13,18-TriHOTrE, was found in roots exposed to the highest dose of TCDD, where their amounts reached 10–12 ng mg^−1^ FW (Fig. 5D). In contrast, the di-hydroxide derivatives of the same fatty acids were relatively less present in the TCDD-exposed roots (Fig. 5D).

Figure 5E summarizes the levels of the specific oxylipin congeners that were most highly induced following administration of a high dose (dark-red) or a low dose (light-red) of TCDD. The dioxin-responsive oxylipins (DROXYL) (18 congeners) can be divided into two main groups (Table 1). Group I contains six oxylipins that were exclusively biosynthesized as a response to TCDD. This includes 7-HpHxTrE, 7-HHxTrE, 10,11,16-TriHHxTrE, 12,13-EpOTrE, 9,10,18-TriHOTrE and 12,13,18-TriHOTrE; Group II consists of twelve oxylipins that were constitutively present at low levels in healthy roots but then upregulated in response to TCDD exposure. Of them, 9-HpOTrE, 9-HOTrE, 9,10-EpHxME, 9,10-EpOME and 9,10-EpOTrE were the most highly stimulated in this group (Table 1). Altogether, these data indicate that the exposure of date palm roots to TCDD stimulates the creation of a specific signature of constitutive and induced of oxylipins suggesting the use of these plant lipids as novel biomarkers for plant exposure to dioxin.

**Table 1:** Dioxin-responsive oxylipins found in date palm roots.

Dioxin-Responsive Oxylipins (DROXYL) in roots of date palm		
Abbreviation	Oxylipin name (IUPAC)	Relevant pathway
**Group I**		
7-HPHT	7-hydroperoxy-hexadecadienoic acid	9-LOX
7-HHT	7-hydroxy-hexadecadienoic acid	PXG
10,11,16-triOH-C16:3	10,11,16-tri-hydroxy-hexadecenoic acid	PXG/ω-CYP450
12,13-epoxy-C18:3	12,13-epoxy-octadecenoic acid	PXG
9,10,18-triOH-C18:3	9,10,18-tri-hydroxy-octadecadienoic acid	PXG/ω-CYP450
12,13,18-triOH-C18:3	12,13,18-tri-hydroxy-octadecadienoic acid	PXG/ω-CYP450
**Group II**		
9-HPOD	9-hydroperoxy-octadecenoic acid	9-LOX
9-HPOT	9-hydroperoxy-octadecadienoic acid	9-LOX
9-HOD	9-hydroxy-octadecenoic acid	PXG
9-HOT	9-hydroxy-octadecadienoic acid	PXG
9,10-epoxy-C16:1	9,10-epoxy-hexadecenoic acid	PXG
7,8-epoxy-C16:3	7,8-epoxy-hexadecadienoic acid	PXG
10,11-epoxy-C16:3	10,11-epoxy-hexadecadienoic acid	PXG
9,10-epoxy-C18:1	9,10-epoxy-octadecenoic acid	PXG
9,10-epoxy-C18:2	9,10-epoxy-octadecenoic acid	PXG
9,10-epoxy-C18:3	9,10-epoxy-octadecadienoic acid	PXG
9,10,16-triOH-C16:1	9,10,16-tri-hydroxy-	PXG/ω-CYP450
9,10,18-triOH-C18:2	9,10,18-tri-hydroxy-octadecenoic acid	PXG/ω-CYP450

### PdPXG2-derived oxylipins can be used as “lipid biomarkers” of dioxin exposure

**Figure 6:**
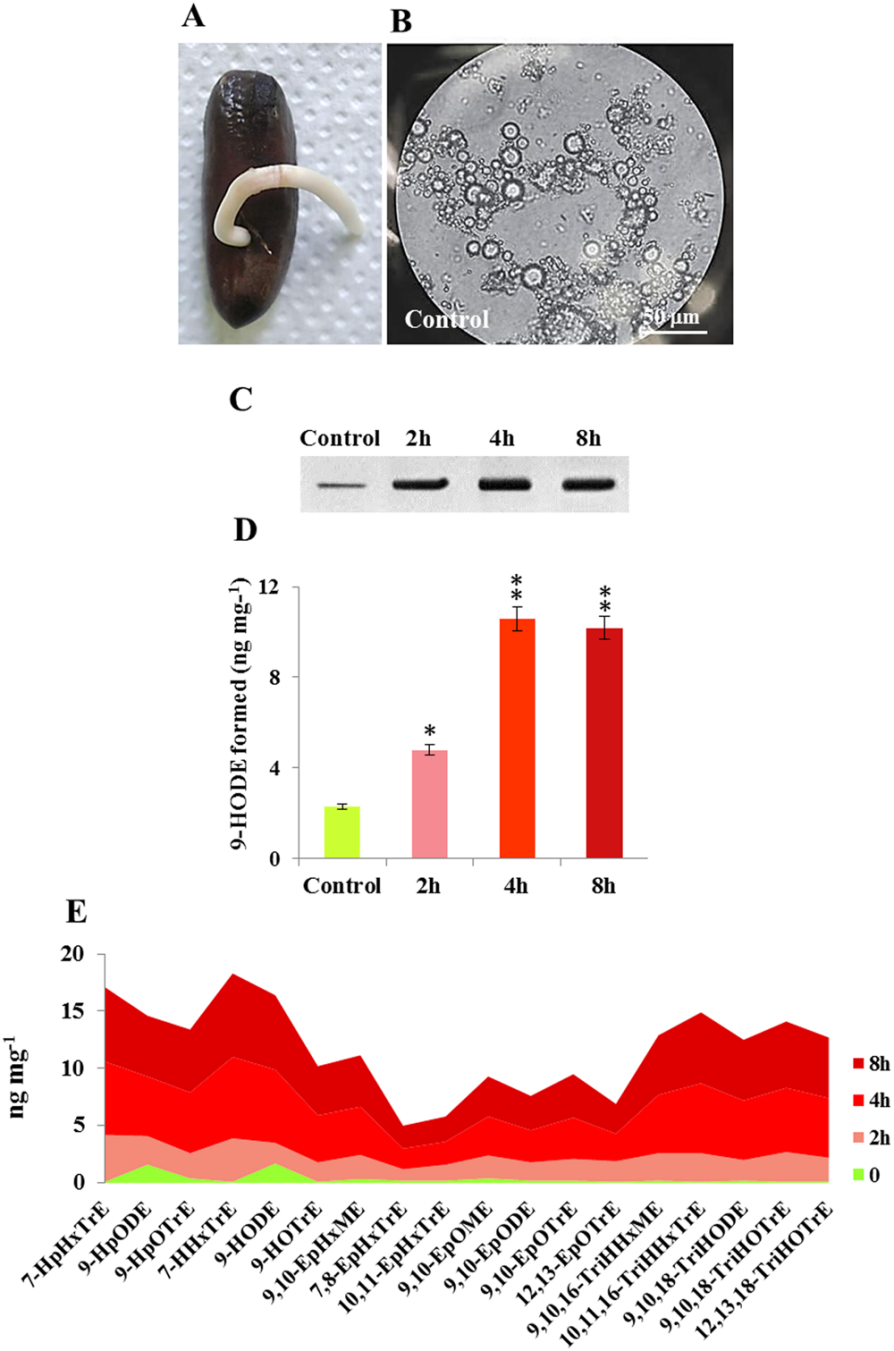
Induction of PdPXG2-derivatives oxylipins in the date palm root-protoplasts after exposure to TCDD. (**A**) Image of a stage I-date palm seedling showed the root that was taken to prepare protoplasts. (**B**) Micrograph of prepared protoplasts. Bar represents 50 μm. (**C**) Immunoblotting of PdPXG2 protein in control protoplasts and protoplasts after exposure to 10 ng L^−1^ TCDD for 2, 4 and 8 h. (**D**) 9-HpODE-reductase activity of PdPXG2 in control protoplasts and TCDD-treated protoplasts for 2, 4 and 8 h. (**E**) Overall evaluation of quantitative areas covered by TCDD-induced oxylipins (18 congeners) after addition of TCDD (10 ng L^−1^) (light-red for 2 h), (red for 4 h) and (dark-red for 8 h), compared with controls (light-green for 0 h). All measurements were done in triplicate. Values are the means ± S.D. (*n* = 3). Asterisks indicate significant differences in the reductase-activity between TCDD-treated and control protoplasts (*P < 0.05; **P < 0.01).

**Figure 7:**
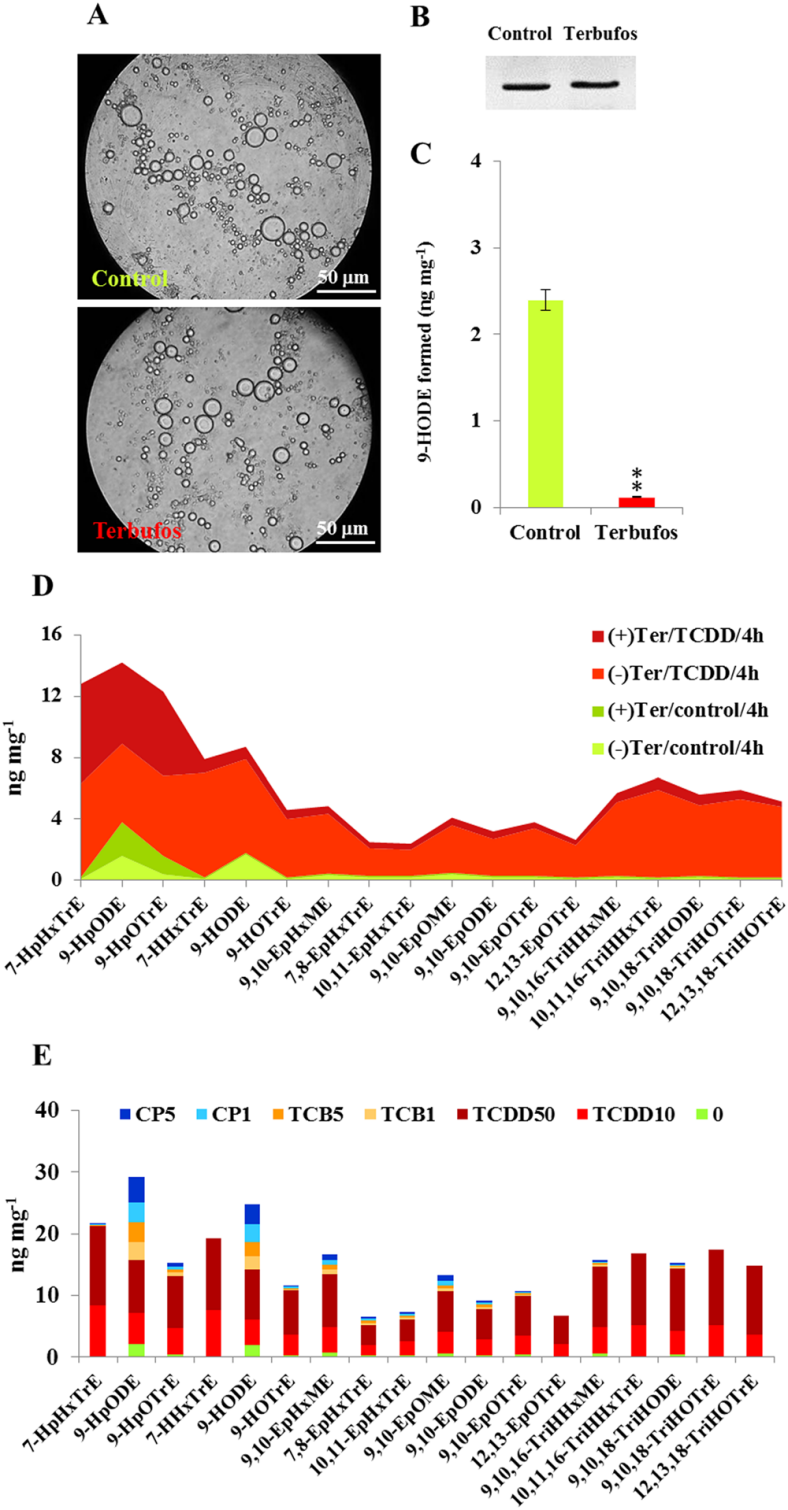
Inhibition of PdPXG2 abolished the induction of TCDD-responsive oxylipins in the root-protoplasts. (**A**) Micrographs of control protoplasts (upper) and protoplasts pre-treated with 1 mM terbufos (Ter) (lower) for 2 h at room temperature. Bar presents 50 μm. (**B**) Immunoblotting of PdPXG2 protein in control protoplasts and in terbufos-pretreated protoplasts. (**C**) 9-HpODE-redyctase activity in control and terbufos-pretreated protoplasts. (**D**) Overall evaluation of the quantitative areas covered by TCDD-responsive oxylipins (18 congeners) in control and in terbufos-pretreated protoplasts after addition of TCDD (10 ng L^−1^) for 4 h. Color coding: light-green for control protoplasts, green for control protoplasts with terbufos, light-red for TCDD-exposed protoplasts without Ter and dark-red for TCDD-exposed protoplasts with Ter. (**E**) Evaluation of the TCDD-specificity of the DROXYL signature in comparison with other types of stressor agents. Similar to what we did for TCDD, the oxylipins signature was determined in the root that treated with 3,3′,4,4′-Tetrachlorobiphenyl (TCB) or with chlorpyrifos-methyl (CP) at two different concentration of each (1 and 5 μg L^−1^ for TCB) and (1 and 5 mg L^−1^ for CP). All measurements were done in triplicate. Values are means ± S.D. (*n* = 3). Asterisks indicate significant differences in the reductase-activity between tebufos-pretreated and control protoplasts (**P < 0.01).

The TCDD-induced profile of oxylipins resulting in the root of date palm was validated in a root protoplast system. To this end, protoplasts were isolated from young roots of date palm (stage I, Fig. 6A), purified and assayed for their vitality (Fig. 6B). When the protoplasts were exposed to 10 ng L^−1^ of TCDD for 2, 4 and 8 h, they accumulated more PdPXG2 protein compared with controls and this accumulation peaked 4 h after exposure (Fig. 6C). In parallel, enzymatic extracts from TCDD-exposed protoplasts more actively metabolized 9-HpODE into 9-HODE compared with controls (10.6 versus 2.3 ng of 9-HODE formed mg^−1^ of protein) (Fig. 6D). The comparative analysis of the eighteen congeners of the TCDD-responsive oxylipins (presented in Fig. 5E) showed a similar profile of such oxylipins in the root protoplasts after exposure to TCDD (Fig. 6E). Well superimposed on their profile *in planta*, both group I and II oxylipins were stimulated in the TCDD-exposed protoplasts and peaked at 4 h and 8 h after exposure. We also evaluated the TCDD-induced oxylipin profile using a specific peroxygenase inhibitor, i.e. terbufos. No cytotoxicity was observed when the isolated protoplasts were pre-subjected to terbufos (Fig. 7A). While no changes were found in the expression of PdPXG2 protein (Fig. 7B), its 9-HpODE-reductase activity was severely reduced (about 20-fold) in protoplasts pre-treated with terbufos (Fig. 7C). Of interest, the PXG-inhibited protoplasts were seriously affected in their ability to respond to TCDD. With the exception of 7-HpHxTrE, 9-HpODE and 9-HpOTrE, all other PdPXG2-derivatives oxylipins (15 congeners) were briefly stimulated by TCDD in the terbufos-pretreated protoplasts when compared to the respective controls (Fig. 7D). Furthermore, the TCDD-specificity of the root oxylipin signature was confirmed when this signature was determined upon the exposure of the date palm root to two types of compounds, the 3,3′,4,4′-Tetrachlorobiphenyl (TCB), an organic environmental contaminant and chlorpyrifos-methyl (CP), an organophosphorus insecticide that has been widely used to control various pests of date palm. Figure 7E shows that the group I of the TCDD-responsive oxylipins was highly specific to the TCDD-exposed root and absent in the TCB- or CP-treated roots. In contrast, levels of 9-HpODE and its corresponding alcohol were increased in response to TCB and CP but less than in response to TCDD, while group II oxylipins were mainly induced in response to TCDD (Fig. 7E). Altogether, these data indicate that PdPXG2-derivatived oxylipins are induced by dioxin and suggest that their presence could be used as signature and an accurate biomarker for plant exposure to this toxin.

### Impacts of PdPXG2 activity on the suberin composition and permeability towards exogenous dioxin of roots

**Figure 8:**
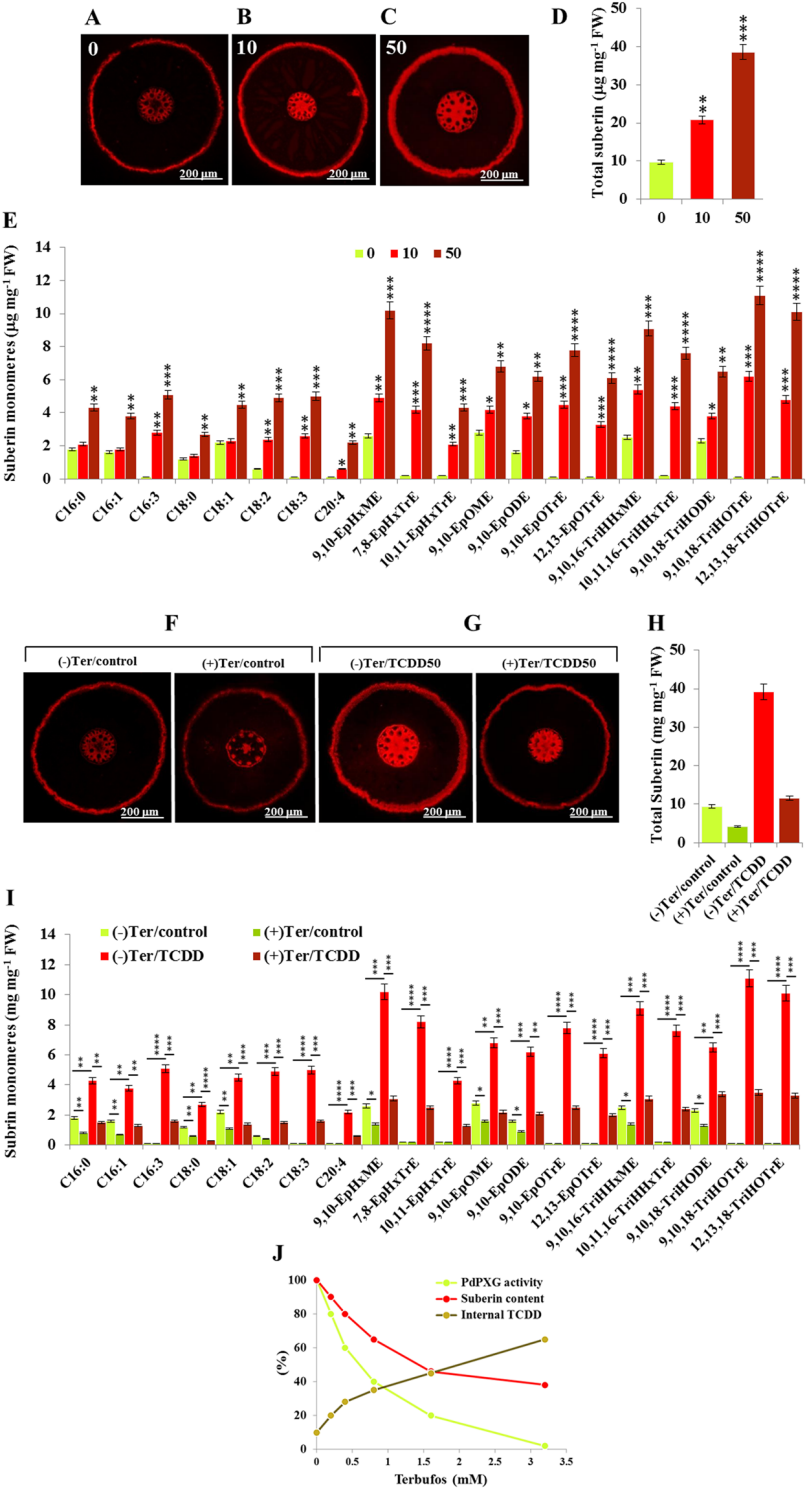
PdPXG2 activity is likely necessary to the deposition of suberin and therefore to their permeability toward dioxin. (**A**–**C**) Micrographs of freehand sections of control date palm roots and roots exposed to TCDD with 10 and 50 ng L^−1^, respectively, under fluorescent microscopy. Bar presents 300 μm. (**D**) Amount of total suberin extracted from TCDD-exposed root at both concentrations compared with control roots. (**E**) Qualitative and quantitative determinations of suberin composition in roots exposed to a low (light-red) and a high dose (dark-red) of TCDD, compared with control roots (light-green). (**F**) Micrographs of freehand sections of control roots without (left) or with Ter (right) and exposed roots with or without Teb. (**G**) Micrographs of freehand sections of TCDD-exposed roots without (left) or with Ter (right). (**H**) Total amount of suberin in control roots without or with Teb (light-green and green columns, respectively) and in TCDD-exposed roots without or with Ter-pretreatment (red and dark-red columns, respectively). (**I**) Composition of suberin in control roots without or with Teb (light-green and green columns, respectively) and in TCDD-exposed roots without or with Ter-pretreatment (red and dark-red columns, respectively). (**J**) Progressive inhibition of PdPXG2 by increasing concentration of Terbufos in correlation with the total amount of root suberin and their permeability against the TCDD. Bar presents 300 μm. All measurements were in triplicate. Data are means ± S.D. (*n* = 3). *P < 0.05; **P < 0.01; ***P < 0.001; ****P < 0.0001.

The effect of increasing accumulation of TCDD-responsive oxylipins on the deposition and composition of root suberin and its subsequent permeability towards TCDD were analysed as shown in Fig. 8. Post-staining of whole roots with neutral red showed that the pigment was concentrated in the walls of epidermal cells and was more intense in TCDD-exposed root than controls (0) with the intensity of fluorescence positively correlated with levels of applied TCDD from 10 to 50 ng L^−1^ (Fig. 8A–C). These observations were confirmed by measuring the amount of suberin in roots. Compared to non-exposed roots, the amount of suberin increased by about 2- and 4-fold in roots exposed to 10 and 50 ng L^−1^ TCDD, respectively (Fig. 8D). Comparative analysis of suberin composition showed a significant increase in certain monomers notably the C16:3, C18:3 and C20:4 as well as the mono-epoxides and tri-hydroxides of C16:3 and C18:3, in roots exposed to TCDD (Fig. 8E). Of these, the 9,10,18-TriHOTrE monomer was the most highly induced by TCDD with about 62- and 111-fold increases in roots exposed to10 and 50 ng L^−1^ TCDD, respectively, compared to controls.

The TCDD-induced enhancement of the overall deposition and the altered composition of root suberin was repressed when roots were pre-treated with terbufos, a specific inhibitor of PXG activity. As shown in Fig. 8F,G, cross-sections of both control and TCDD-exposed roots showed much less suberin was present when they were pre-treated with terbufos. The inhibition of PXG activity by terbufos caused a net decrease in the total suberin amount of about 2.2-fold in the root control and about of 3.5-fold in TCDD-exposed roots (Fig. 8H). The quantitative reduction in suberin composition was confirmed by a comparative analysis of suberin monomers as shown in Fig. 8I. Root permeability to TCDD was assessed by measuring its concentration inside roots. A positive linear relationship between the deposition of root suberin and root permeability towards TCDD was found. The decreasing deposition of suberin, due to the inhibition of PdPXG2, was correlated with an increasing permeability to TCDD and vice versa (Fig. 8J). These data suggest that the response of date palm roots to TCDD occurs due to enhanced deposition of suberin with an altered FA composition and this is probably mediated by the concurrently induced PdPXG2 activity.

## Discussion

Dioxins are amongst the most hydrophobic environmental contaminants, which makes them extremely toxic when they are taken up into biological systems. Due to their high lipophilicity, dioxins mainly accumulate in fat-rich tissues and cause various forms of metabolic damage. Plants grown in contaminated environments can take up dioxins via their roots from which they can be transported into aerial plant tissues such as leaves and seeds, thereby affecting the lipid status by modulating enzymes including some LD-associated enzymes, especially caleosin/peroxygenases^22,23,38^.

Recently, two caleosin/peroxygenases, PdPXG2 and PdPXG4, were characterized in date palm seedlings regarding their tissue expression, subcellular localisation and enzymatic activities. Following exposure to TCDD, PdPXG2 was more highly expressed in roots of date palm seedlings and showed a pronounced specificity in reducing the 9-hydroperoxide of C18:2 (9-HpODE)^43^. In this study we describe the involvement of this peroxygenase in the root response to dioxin exposure via its highly distinctive enzymatic activity. The PdPXG2 protein has similar structural and biochemical properties to those found in previously characterized plant caleosin/peroxygenases, e.g., a Ca^2+^-binding EF-hand motif, a central hydrophobic domain including a proline knot, two invariant histidine residues responsible for heme/iron binding and several putative phosphorylation sites^32,33,39,45–48^. However, this date palm peroxygenase also has highest similarity to two Arabidopsis caleosin/peroxygenases, AtPXG5 and AtPXG7, that have yet to be physiologically characterized and these may be members of a new sub-class of PXGs with novel functions, especially regarding responses to certain abiotic stresses. The small but distinctive divergence in amino acid sequences between these PXGs and previously studied PXGs is reflected in the unusual catalytic activity of PdPXG2, which preferably reduces 9-hydroperoxide fatty acids, in contrast to PXGs in Arabidopsis, oat, rice and date palm tissues that preferably reduced 13- hydroperoxide fatty acids^27,32,33,43^. The date palm PdPXG2 also has a low epoxidation activity compared to other plant PXGs, which may be due to it being mainly expressed in root tissues, unlike other plant PXGs that are mostly leaf expressed where their epoxide products are implicated in cuticle formation^32,34,49–51^. Indeed, plant PXGs were originally found to be most actively expressed in aerial tissues of young seedlings of Arabidopsis, oat, rice and soybean^32,49–52^. In contrast, we found that PdPXG2 was mainly expressed, in terms of transcript and protein abundance and enzyme activity levels, in young roots and especially in the apical zone where its expression was strongly induced after TCDD exposure. Such a specific tissue localisation in an apical meristematic zone is interesting in view of recent findings of the high expression of caleosins in the shoot tip transcriptome of male willow plants^53^. To our knowledge, the current study is the first report of a 9-LOX/PXG pathway in plant roots.

Other evidence shows that 9-LOX derivative oxylipins play pivotal roles in plant defense against pathogens^54–57^. It was suggested that 9-LOX-derived oxylipins can act as regulator signals of programmed cell death^58,59^, as antimicrobial molecules, or in cell wall modification via a (+)−7-iso-jasmonic acid-independent pathway^35,55,60^. It was also reported that 9-LOX regulates the response to lipid peroxidation-induced singlet oxygen formation, suggesting a role for the 9-LOX pathway in the modulation of oxidative stress, lipid peroxidation and plant defense^57,61^. In addition to their roles as stress-responsive molecules, 9-LOX oxylipins are implicated in many plant developmental processes. For example, a specific 9-LOX-encoding gene is transiently induced during potato tuber filling and its antisense suppression resulted in reduced tuber size^62^. In this context, our analysis showed that the PdPXG2-derived oxylipins are most abundant in date palm roots. Several lines of evidence suggest that 9-LOX-derived oxylipins have roles in root development. For example, maize *9-LOX* knockout mutants displayed precocious senescence and much reduced root length^63^. Likewise, we previously found that TCDD-exposed Arabidopsis plants had high levels of LOX in their lateral root systems^22^. Furthermore, studies with *noxy2* (for non-responding to oxylipins2), a new 9-HOTrE–insensitive mutant, has shown that exogenous application of 9-HOTrE (9-hydroxy-10,12,15-octadecatrienoic acid) to Arabidopsis plants resulted in root abnormalities such as lateral root arrest, overall growth arrest, loss of root apical dominance and increased plant tolerance to ROS^60,64^. Moreover, a 9-LOX-derived analog to OPDA, 10-OPEA (10-oxo-11-phytoenoic acid), has been shown to minimize the infection of maize roots by root-knot nematodes^63^.

More recently, it was demonstrated that 9-LOX-derived oxylipins induce brassinosteroids (BRs), a class of plant hormones necessary for normal plant growth and the control of cell wall integrity, suggesting a sequential action of 9-LOX and BR signaling in activating cell wall-based defense, such as callose deposition, as part of the prevention of pathogen infection^65^. The possible involvement of 9-LOX oxylipins in regulating cell wall composition is in line with our data showing that part of the PdPXG2 response to TCDD involves the modulation of suberization of cell walls, thereby modifying their permeability toward the dioxin. This was demonstrated using a specific inhibitor of PdPXG2 activity and by histological analysis of root tissues.

Due to the lipidic nature of suberin monomers^66,67^, stress-induced alternations in the lipid metabolism could lead to modifications to overall suberin composition. This is borne out by data from several lipid metabolism mutants of Arabidopsis that are specifically affected in suberin composition and also have severe deficiencies in their responses to biotic and abiotic stress. For example, at least two acyltransferases, GPAT4 and GPAT8, are required for the synthesis of suberin in the seed coat and roots of Arabidopsis^68,69^. Also, the mutants *horst-1* and *horst-2* that contain knockout alleles of cytochrome P450 fatty acid ϖ-hydroxylase CYP86A1 (At5g58860) were characterized by a dramatic reduction in the total aliphatic monomers of root suberin^70^. Moreover, non-specific lipid transfer proteins (nsLTPs), facilitating phospholipid transfer between membranes *in vitro*, play a potential role in suberin formation^71^. Particular support for our data comes from reports on the involvement of a PXG pathway in the biosynthesis of cutin monomers, where the specific inactivation of the PXG by terbufos resulted in a dramatic decrease in cuticular epoxide content and reduced cuticle thickness in maize plants where these effects were restricted to plants containing cutin originating from C18^34,51,72^. It has also been suggested that the Arabidopsis epoxide hydrolase1 (AtEH1), a cytosolic enzyme that hydrolases the epoxy-fatty acids formed by the PXG, is involved in the synthesis of polyhydroxylated cutin monomers^73^. The increase in root permeability to dioxin due to the inhibition of PdPXG2 reported here is also supported by the fact that the composition and deposition of suberin are modulated in response to unfavourable environmental conditions such as drought and salt stress^74–78^ as well as to wounding^79,80^. Such alternations in suberin status have been shown to affect the movement into plants of water and dissolved nutrients^81–85^, plant responses to root pathogens^86–90^, and root permeability to pesticides^51^.

Finally, our data indicate that exposure of date palm roots to TCDD leads to the formation of a specific signature of constitutive and induced of oxylipins, especially poly-hydroxy fatty acids, suggesting a particular involvement of this group of plant oxylipins as “biomarkers” for plant exposure to dioxins. Of particular interest, one of the biological implications of increasing levels of PUFAs is shown by the modification in the composition of cell membrane FAs under the activation of adjacent membrane-bound desaturases that can modulate membrane permeability and acclimation of plants to changing environmental conditions^91,92^. Although there are no comparative data on the effect of persistent xenobiotics such as dioxins on the plant lipidome, similar lipidic responses were reported in dioxin-exposed marine animals^93,94^. Therefore, we suggest that such a lipidome “signature” could be used as a biomarker to assess the severity of animal exposure to dioxins in an analogous manner to our suggestion here for plants^95,96^.

## Conclusion

We describe a novel isoform of caleosin/peroxygenase, PdPXG2, that is mainly expressed in the roots of date palm seedlings and specifically reduces 9-hydroperoxide fatty acids. This peroxygenase is induced in roots following administration of exogenous TCDD, the most toxic congener in the dioxin group, and its activity leads to the creation of a specific “signature” of dioxin-responsive oxylipins. The accuracy of this lipidic signature was validated in term of quality and quantity *in planta* as well *in vitro*. This suggests the use of dioxin-responsive oxylipins as biomarkers for monitoring the exposure of plants and animals to dioxins.

## Methods

### Plant materials, culture conditions and TCDD-treatment

Date palm (*Phoenix dactylifera* L.) seeds were collected from the fruits of the Khalas cultivar, washed, air-dried and stored in plastic bags at room temperature. Seeds were germinated *in vitro* as described previously. Briefly, seeds were placed into a current of running water for two weeks before then the intumescent seeds were sown onto two layers of gauze and covered with two further layers of a solidified-plastic transparent box (20 × 13 × 8 cm). Cultures were placed in an incubator at 30 ± 2 °C and humidified daily. Seedlings with a radicle length of 0.5, 2.5, 5, 8 and 13 cm were referred as stage I, II, III, IV and V, respectively. The 2,3,7,8-tetrachlorodibenzo-p-dioxin (2,3,7,8-TCDD dissolved in toluene at concentration of 10 μg mL^−1^, purity 99%) was purchased from Supelco Inc., USA. For TCDD-treatments, the preparation of the initial solution of TCDD was done as follows: the TCDD (10 μg) was taken in 10-mL capped glass tubes and evaporated to dryness under a flow of nitrogen. For health and environmental safety reasons, residual TCDD was re-dissolved in a minimum volume (100 μL) of dimethyl sulfoxide (DMSO) in a 15 mL polypropylene test tube, and 5 mL of aqueous solutions of TCDD were prepared in deionized and distilled water to obtain final concentrations of 0, 10 and 50 ng L^−1^ TCDD. 3,3′,4,4′-Tetrachlorobiphenyl (3,3′,4,4′-TCB) and chlorpyrifos-methyl (O,O-diethyl O-3,5,6-trichloro-2-pyridyl phosphorothioate, CP) were purchased from Sigma-Aldrich (USA) and dissolved in DMSO as described before to obtain concentrations of 1 and 5 μg L^−1^ for TCB or 1 and 5 mg L^−1^ for CP. Seedlings were irrigated with indicated TCDD concentrations twice a week. Responses to TCDD were analysed in the roots during developmental stages I, II, III, IV and V. For each stage, root was divided into sections. More detailed information and descriptions on the age, length for each stage of the root development as well as the number of section and their length are summarized in Table S1. For each development stage, six seedlings were used and measurements carried out in triplicate.

### Preparation of protoplasts from date palm root

Protoplasts were isolated as described by Chen and Halkier^97^ and modified by Zhai *et al*.^98^. Briefly, two grams of root in the stage III were sliced with a razor blade to 1-mm pieces and immediately placed into 10 mL of filter-sterilized solution of 0.3 M sorbitol and 50 mM CaCl_2_. Twenty millilitres of enzyme solution [0.5 M Sucrose, 10 mM MES-KOH, 20 mM CaCl_2_, 40 mM KCl, 1% Cellulase R-10 (Sigma-Aldrich), 1% Pectinase R-10 (Sigma-Aldrich), pH 5.7] were added to the root pieces, and the mixture agitated at 30 rpm in the dark at room temperature for overnight. The released protoplasts were filtered through five layers of cheesecloth, suspended into 10 mL of suspension solution (0.1% [w/v] Glucose, 0.08% [w/v] KCl, 0.9% [w/v] NaCl, 1.84% [w/v] CaCl_2_, 2 mM MES-KOH, pH 5.7). Protoplasts and plant debris retained on the filter-cloth were washed with 10 mL of suspension solution. The protoplasts were combined in a 50-mL Falcon centrifuge tube, overlaid with 10 mL of suspension solution, and centrifuged for 5 min at 2000 rpm. Protoplasts were collected at the interface of enzyme and suspension solutions and their yield was evaluated by cell counting with a hemocytometer.

### Analysis of genes transcripts

Changes in relative transcriptional abundance of *PdPXG2* gene in response to TCDD exposure were analysed by reverse-transcription quantitative PCR (RT-qPCR) as described^43^. For RNA extraction, one gram of frozen fine powder taken from roots at stages I, II, III, IV and V was used to extract total RNA using an RNeasy kit according to the manufacturer’s instructions (Qiagen, Germany). Aliquots of 1 μg total RNA were used for first-strand cDNA synthesis according to Hanano *et al*.^22^. Real-time PCR was performed in 48-well plates using a AriaMx Real-time PCR System from Agillent technologies, USA. In brief, 25 μL reaction mixtures contained 0.5 μM of each specific oligonucleotide primer for the target (*PdPXG2*) (GGCGTCCTCATCGTTACCTT/GTTCCGGTCAAAGAAGGCGA) and reference (*TIP-41*) (GAACTGGCTGACAATGGAGTG/ATCAACTCTCAGCCAAAATCG), genes 12.5 μL of SYBR Green PCR mix (Bio-Rad, USA) and 100 ng cDNA. The relative quantification RQ of target genes was calculated using the software installed in the qPCR system. Sequences of amplified regions were confirmed on an ABI 310 Genetic Analyzer (Applied Biosystems) using a Big Dye Terminator kit (Applied Biosystems).

### SDS-PAGE and Western blotting

Microsomes and lipid droplets (LDs) were isolated from roots of date palm seedlings at stage III as previously described^32^. Briefly, washed microsomes or LDs (30 mg protein) were resuspended in 5 ml of a 10 mM Tris-HCl buffer (pH 8) containing 10% glycerol (buffer A) and solubilised with 0.2% (v/v) emulphogene (polyoxyethylene 10 tridecyl ether, Sigma-Aldrich) for 45 min at 4 °C^32^. Solubilised proteins were quantified by a Bradford assay (Bio-Rad) using bovine serum albumin as a standard^99^. Proteins were analyzed by SDS–PAGE using 12% polyacrylamide gels and electroblotted onto a PVDF membrane (Millipore) in a Semi-Dry Transfer Cell (Bio-Rad). PdPXG2 was immunodetected by incubating membranes with a polyclonal antibody prepared from the complete sequence of the AtClo1 caleosin isoform from *Arabidopsis thaliana*, as described (Hanano *et al*.^32^). After protein transfer, the membranes were blocked overnight at 4 °C in a solution of 3% (w/v) bovine serum albumin (BSA) in Tris-buffered saline (TBS), pH 7.4. Then, the membranes were incubated overnight within a 1:2000 dilution of a AtClo1-antibody prepared in TBS-T buffer containing 0.3% (v/v) Tween-20 at 4 °C. After three washes in TBS-T for 5 min each. The membranes were incubated within a 1:5000 dilution of a horseradish peroxidase conjugated goat anti-mouse secondary antibody (sigma-Aldrich) for 1 h at room temperature. After three washes in TBS-T as before, the bands were colorimetrically detected by adding 3-amino-9-ethylcarbazole (Sigma-Aldrich), as a chromogenic substrate, in acetate buffer and in the presence of a hydrogen peroxide. Membranes were manually photographed using a camera (Olympus FE4000, 12 Mega Pixel).

### Enzymatic assays

The epoxidation activity of PdCOL2 was assayed according to Blee and Durst^52^ by incubating 50 μg of purified recombinant protein with 100 μM of a given [l-^14^C]-labeling fatty acid (Table S2) and 100 μM cumene hydroperoxide or 13-hydroperoxylinoleic acid in 100 μL of Buffer A, i.e. 10 mM sodium acetate buffer (pH 5.5) containing 20% (v/v) glycerol and 0.1% emulphogene at 25 °C for 5 min. The reaction was stopped by addition of 0.1 mL of acetonitrile containing 0.2% acetic acid. For monounsaturated fatty acids (C14:1, C16:1 and C18:1), the totality of each reaction mixture was separately spotted onto a C_18_ reversed-phase TLC plate (Aluminum sheets 20 × 20 cm, 200 μm layer, Merck, Germany) and developed in a diethyl ether/petroleum ether/formic acid (50/50/1, v/v/v) solvent system. Radioactivity of the bands corresponding to the residual fatty acids and their respective epoxides was then determined.

For polyunsaturated fatty acid (C18:2, C18:3 and C20:4), the substrates and products of each reaction were extracted twice with 2 volumes of ethyl ether, and evaporated to dryness under a stream of argon. Methyl ester derivatives were produced using diazomethane, separated on TLC with hexane/ethyl acetate (85/15, v/v). Bands corresponding to mono-epoxide and di-epoxide methyl esters of fatty acids, which co-migrated with authentic standards, were scrapped off and analyzed by GC and GC/MS as described below. Hydroperoxide-reductase activity was measured by incubation of 9-HpODE, 13-HpODE 9-HpOTrE and 13-HpOTrE overnight at 26 °C with 50 μg of purified recombinant proteins in 500 μL of sodium acetate (0.1 M, pH 5.5). Residual substrate and products were extracted in 3 × 2 mL of dichloromethane/ether (1/1, v/v). After drying under nitrogen flow, extracts were taken with 25 μL of acetonitrile/water/acetic acid (50/50/0.1, v/v/v).

### Extraction and preparation of root oxylipins

Extraction of total oxylipins from date palm roots was carried out as described^100^. In brief, two grams of frozen roots taken at various developmental stages (I, II, III, IV and V) was firstly ground into the liquid nitrogen then transferred to a 50-mL glass tube plus 10 mL of extraction solvent [isohexane/2-propanol, 3/2 (v/v), 50 mg butylated hydroxytoluene (BHT) and 15 mg triphenylphosphine (TPP)]. After homogenization on ice for 5 min, the extract was centrifuged at 1300 × *g* at 4 °C for 10 min. The organic upper phase was recovered and a 6.7% (w/v) solution of potassium sulfate was added to obtain a volume of 16 mL. After vigorous shaking, the extract was re-centrifuged. The upper hexane-rich layer containing the free fatty acid derivatives oxylipins was collected, its volume was reduced to 0.5 mL under a flow of nitrogen and then stored at 4 °C for further analysis. The following internal standards were used: (18:0, 18:0) MGDG and (18:0, 18:0) DGDG (5 mg each) and (6Z, 9Z, 11E, 13 S)-13-Hydroxy-6,9,11-octadecatrienoic acid (300 ng) (Sigma-Aldrich, USA). For analysis of esterified oxylipins, transmethylated with sodium methoxide was done as described^59^. After evaporation of extracts under nitrogen, 333 μL of a methanol/toluene (2/1) solution and 167 μL 0.5 M CH_3_NaO were added to the residual oxylipins and the mixture was agitated for 20 min at room temperature. A saturated NaCl solution (500 μL) and 20 μL of 32% HCl (v/v) were then added. Subsequently, the sample was extracted twice with an equal volume of *n*-hexane (v/v), and the organic phase was taken and dried under nitrogen. Fatty acid hydroperoxides were extracted from plant tissues and analyzed according to Göbel *et al*.^54^. Two grams of plant tissues were ground in liquid nitrogen. After adding 10 mL of extraction solvent (*n*-hexane:2-propanol, 3/2 (v/v) with 0.025% (w/v) butylated hydroxytoluene), the mixture was immediately ultra-homogenized for 30 s on ice. A spiked sample with 10 μM of each hydroperoxide was used as a control. The extract was shaken for 10 min and centrifuged at 3,000 × *g* at 4 °C for 10 min. The upper organic phase was carefully taken and a 6.7% (w/v) solution of potassium sulfate was added up to a volume of 16.2 mL. After vigorous shaking and a brief centrifugation at 4 °C, the upper layer was taken and dried under streaming nitrogen. Extracted hydroperoxides were taken within 25 μl of acetonitrile/water/acetic acid (50/50/0.1) (v/v/v) and their quantification were carried out on a Jasco LC-2000 plus series HPLC system (Jasco, USA) as described below. Three samples of each stage were taken, three separate and parallel extractions were done for each sample and three measurements were performed for each extraction.

### Analytical methods

Radioactivity was determined on TLC plates by a Berthold TLC linear analyzer LB 283 and peaks integrated by a data acquisition system LB 511. Spectrophotometric measurements were done on a Shimadzu model MPS-2000 spectrophotometer. Capillary gas chromatography analyses of methyl esters of monounsaturated or polyunsaturated fatty acids and their epoxide derivatives were performed on an Agilent Technologies 7890 GC System (USA) coupled to an AMD 402 high resolution mass spectrometer (Germany). A 5-μL aliquot of the sample was injected into an OVI-G43 capillary GC Column (30 m L × 0.53 mm ID × 3.00 μm d_f_) (Sigma-Aldrich) with helium as carrier gas at a constant flow rate of 1.6 mL min^−1^. The oven temperature program was as described by Blee and Durst^52^ as follows: start at 150 °C, hold for 1 min, increase to 200 °C at 30 °C min^−1^, increase to 240 °C at 5 °C min^−1^ and hold for 8 min. For separation of multiple monoepoxides and identification of the trihydroxy derivative of a given fatty acid, a fused silica capillary column (30 m L × 0.25 mm ID × 0.25 μm d_f_) coated with DB-Wax (Agilent J&W GC column) was used with the temperature program: 10 °C/min to 150 °C, followed by 3 °C/min to 190 °C and 1 °C/min to 220 °C. Hydroperoxides (-OOH) fatty acids and their respective hydroxides (-OH) were analysed using a Jasco LC-2000 plus series HPLC system (Jasco, USA) with a UV-detector (RF-10Axl, Shimadzu) (234 nm) and a C18 column (Eclipse XDB-C18 150 × 4.6 mm, 5 μm; Agilent, USA). The analysis was performed using a mobile phase of acetonitrile/water/acetic acid (50/50/0.1, v/v/v) at a flow rate of 0.6 mL min^−1^. FA-hydroperoxides were quantified using their respective standards.

### Biochemical characterization

*Heme content-* The heme staining procedure was carried according to^32^. Hemin (Sigma-Aldrich) was used as standard for the quantification of heme at 370 nm. *Phosphorylation*- Proteinase-treated microsomes or LDs were incubated in the presence of 0.1 unit of casein kinase II and 4 μCi of [γ-^35^S]ATP in 100 mM KH_2_PO_4_ buffer (pH 7.5) containing 8 mM MgCl_2_ for 4 h at 27 °C. Proteins and peptides separated by SDS/PAGE were transferred to a polyvinylidene difluoride membrane for immuno- and radio-detections. *Inhibition-* the peroxygenase (PXG) activity was inhibited by incubation of the purified recombinant protein with 1 mM of terbufos or 1 mM of b-mercaptoethanol, known as suicide substrate or competitive Inhibitor, respectively^51^ for 1 h on ice. Protoplasts were pre-treated with 2 mM of terbufos for 2 h at room temperature before exposure to TCDD. *Calcium ions removal*- Removal of Ca^2+^ ions from proteins was carried out as described^32^. In a hemolysis glass tube, 100 μg of purified proteins were incubated with 400 μL of guanidine-HCl solution (6 M) and 100 μL of ethylenediaminetetraacetic acid (EDTA) solution (10 mM) at 4 °C for 4 hours. In a cold chamber, the mixture was dialyzed overnight against l L of EDTA (5 mM, pH 7) followed by dialysis against 1 L of deionized water for 3 hours. The reconstitution of the protein with Ca^2+^ ions was done by incubating the Ca^2+^-depleted protein with 1 mM of CaCl_2_ solution at 4 °C for 1 hour. The protein concentration and enzymatic activity were determined before and after removal and reconstitution of Ca^2+^ ions.

### Histochemical analysis

Freehand cross-sections were made with a razor blade from healthy and TCDD-exposed roots of date palm seedlings. Sections were fixed overnight in a formaldehyde/acetic acid/alcohol 10/5/85 (v/v) solution at 4 °C. Sections were stained for 1 min in a fresh solution of 0.1% (w/v) neutral red (Sigma-Aldrich) in 0.01 M phosphate buffer (pH 6.5), which specifically dyes suberin in epidermal cell walls. Sections were then examined under a Nikon Eclipse Ti-U fluorescent microscope and micrographs recorded at a magnification of 10× a using a Nikon Ti-U camera.

### Statistics

All data were randomly analysed in a blinded manner. Data were expressed as means ± standard deviation (SD) of at least three independent experiments. Two-tailed Student’s t-test or two-way ANOVA were used to evaluate the statistical differences. Statistical significance was estimated when *P* < 0.05.

## Electronic supplementary material

Supplementary information
