# Supplementary material for: Identification of a dioxin-responsive oxylipin signature in roots of date palm: involvement of a 9-hydroperoxide fatty acid reductase, caleosin/peroxygenase PdPXG2
Source: Sci Rep. 2018 Sep 4;8:13181. doi: 10.1038/s41598-018-31342-4 (PMC6123484; doi:10.1038/s41598-018-31342-4)
Supplement: Supplementary file 1 — Supplementary information [file 41598_2018_31342_MOESM1_ESM.pdf]

Identification of a dioxin-responsive oxylipin signature in roots of date palm: involvement of a 9-hydroperoxide fatty acid reductase, caleosin/peroxygenase PdPXG2

Abdulsamie HANANO[1*], Mouhnad SHABAN[1], Ibrahem ALMOUSALLY[1] and Denis J MURPHY[2]

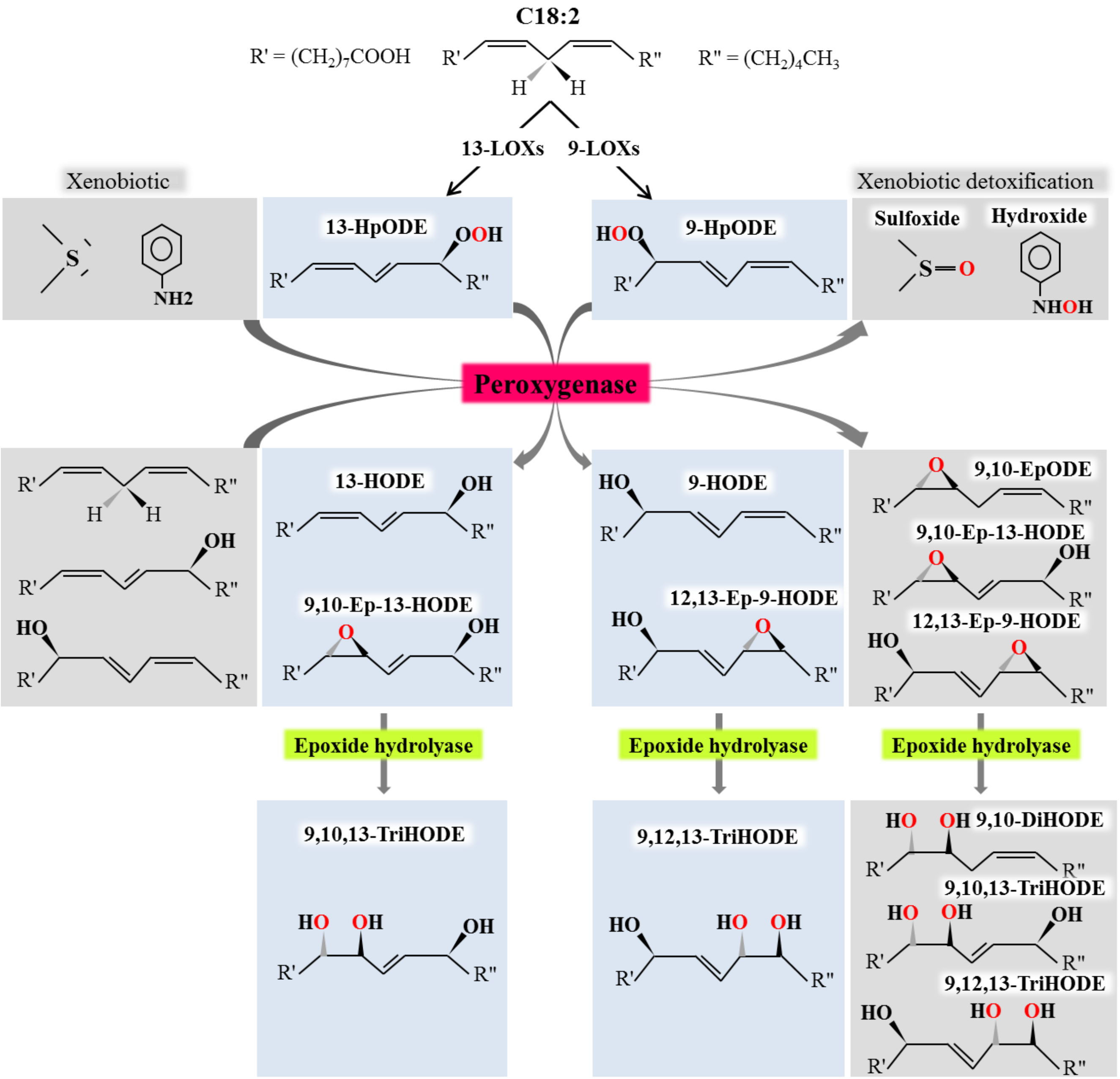

Figure S1: Linoleic acid (C18:2)-derived oxylipins possibly formed under the action of plant Peroxygenase pathway. C18:2 hydroperoxides [13-hydroperoxy-octadecadieonic acid (13-HpODE) or 9-hydroperoxy-octadecadienoic acid (9-HpODE)], formed by 13- or 9-lipoxygenase enzymes (13-LOX or 9-LOX), respectively, are reduced by the peroxygenase (PXG) to corresponding hydroxides [13-hydroxy-octadecadienoic acid (13-HODE) or 9-hydroxy-octadecadienoic acid (9-HODE)], with a possible intra-molecular transfer of oxygen to produce the corresponding epoxy-hydroxy-metabolites [9,10-epoxy-13-hydroxy-octadecadeinoic acid (9,10-Ep-13-HODE) or 12,13-epoxy-9-hydroxy-octadecadeinoic acid (12,13-Ep-9-HODE). In parallel, PXG catalyzes co-oxidation reactions, where oxygen transfer takes place on xenobiotic substrates (sulfuric or aromatic compounds) to produce the corresponding sulfoxide or hydroxides or on biological lipid substrates including C18:2 or its hydroxide resulting corresponding epoxy or epoxy-hydroxy-C18:2. Downstream of PXG, an Epoxide Hydrolyase (EH) can intervene to hydrolyze the epoxide ring to produce the corresponding alcohols, e.g., [9,10,-dihydroxy-octadecadeinoic acid (9,10-DiHODE) or 9,10,13-trihydroxy-octadecadeinoic acid (9,10,13-TriHODE)].

Table S1 Information on the root and their respective tissues used in the study.

Stage	Age/length (days/cm)	Section/length (name/cm)
**I**	15/1.0	S1/1
**II**	25/2.5	S1/1.25, S2/1.25
**III**	35/5.0	S1/1.6, S2/1.6, S3/1.6
**IV**	42/8.0	S1/2, S2/2, S3/2, S4/2
**V**	55/13	S1/2.6, S2/2.6, S3/2.6, S4/2.6, S5/2.6

Table S2 Fatty acids used in the epoxidation activity**.**

**Fatty acid***	***C:D***	**Δ^x**	***n-x***
Myristoleic acid	14:1	cis-Δ^9	*n*-5
Palmitoleic acid	16:1	cis-Δ^9	*n*-7
Palmitelaidic acid	16:1	trans-Δ^9	*n*-7
Hexadecatrienoic acid	16:3	cis,cis,cis-Δ^7,Δ^{10},Δ^{13}	*n*-3
Oleic acid	18:1	cis-Δ^9	*n*-9
Elaidic acid	18:1	trans-Δ^9	*n*-9
Vaccenic acid	18:1	trans-Δ^{11}	*n*-7
Linoleic acid	18:2	cis,cis-Δ^9,Δ^{12}	*n*-6
Linoelaidic acid	18:2	trans,trans-Δ^9,Δ^{12}	*n*-6
Linolenic acid	18:3	cis,cis,cis-Δ^9,Δ^{12},Δ^{15}	*n*-3
Arachidonic acid	20:4	cis,cis,cis,cis-$\Delta^5\Delta^8$,Δ^{11},Δ^{14}	*n*-6

* Common name of the fatty acids

C:D Lipid numbers where *C* is the number of carbon atoms and *D* is the number of double bonds in the fatty acid.

Δ^x indicates where the double bond is located on the xth carbon–carbon bond, counting from the carboxylic acid end.

n-x indicates to double bond is located on the xth carbon–carbon bond, counting from the terminal methyl carbon (designated as *n*) toward the carbonyl carbon.

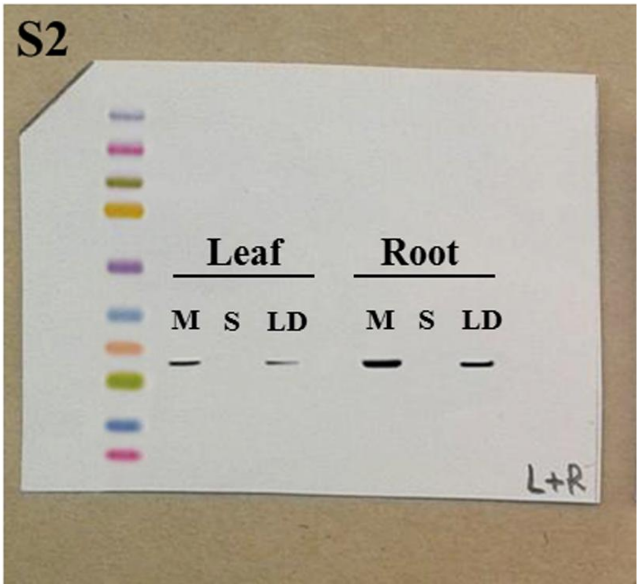

Figure S2: Representative full size image of western blotting membrane presented in the Figure 1D. Immunoblotting of PdPXG2 protein in microsome (M), soluble (S) and lipid droplet (LD) subcellular fractions isolated from leaf and root of date palm seedlings, respectively.

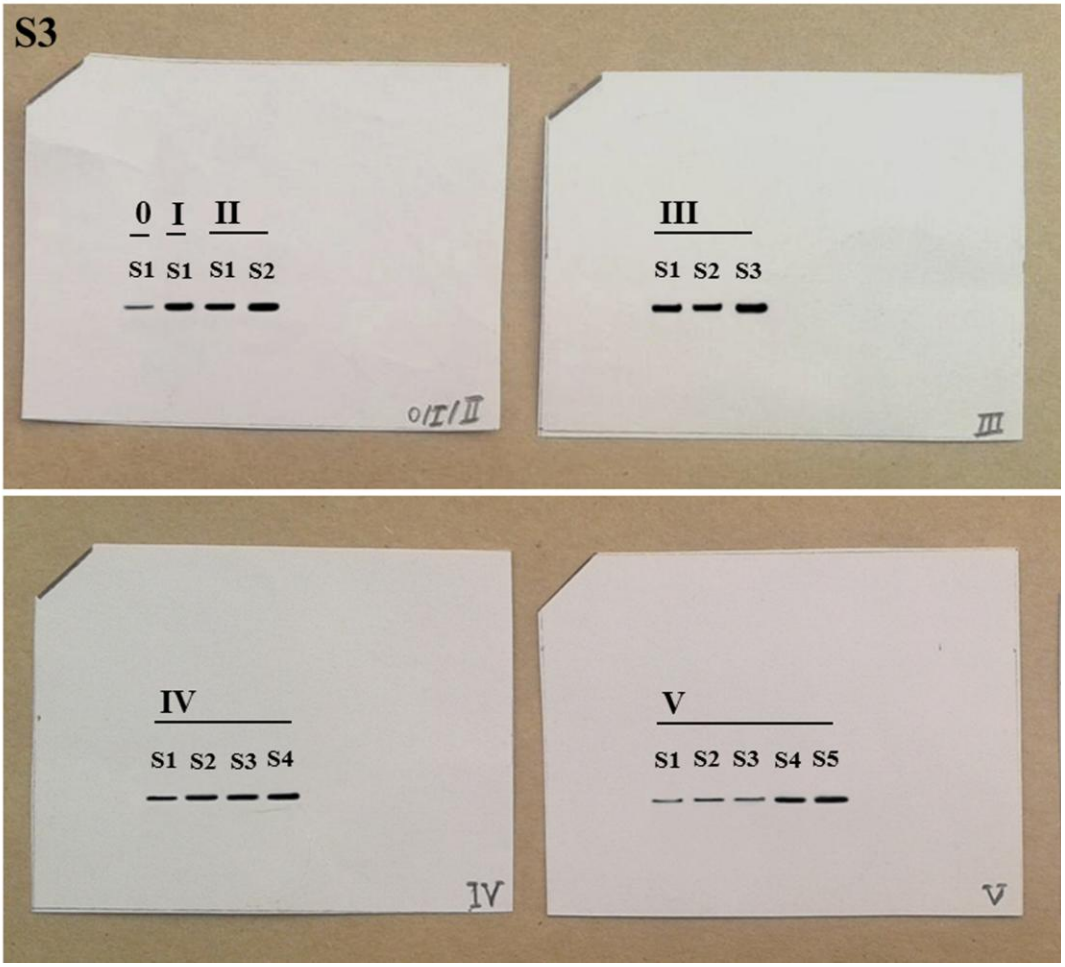

Figure S3: Representative full size images of western blotting membranes presented in the Figure 3C. Immunoblotting of PdPXG2 protein in the whole root of date palm seedlings at different stages of development (0,I, II, III, IV and V) and in different sections for each root from the top to the apical (S1 to S5).

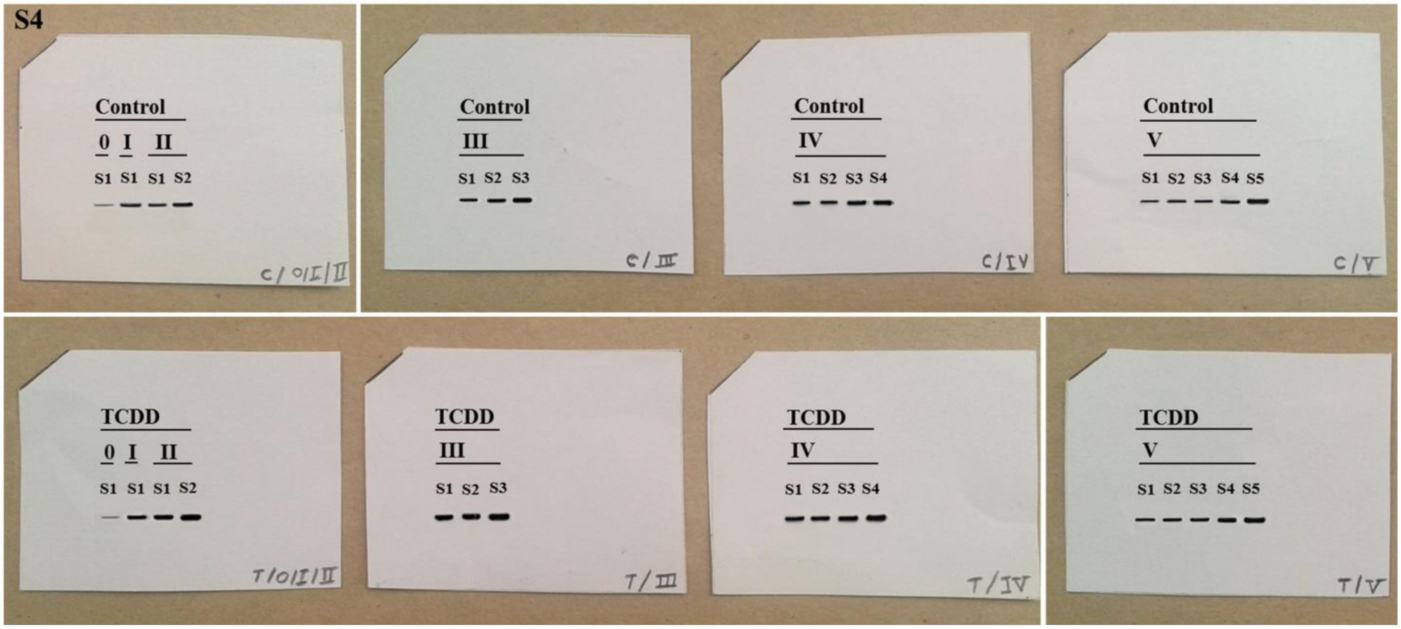

Figure S4: Representative full size images of western blotting membranes presented in the Figure 4B. Immunoblotting of PdPXG2 protein in the root of date palm seedlings of control samples and those treated with 50 ng.L^{-1} as a function of section localization from the top to the root apical (S1 to S5) at stages 0, I, II, III, IV and V.

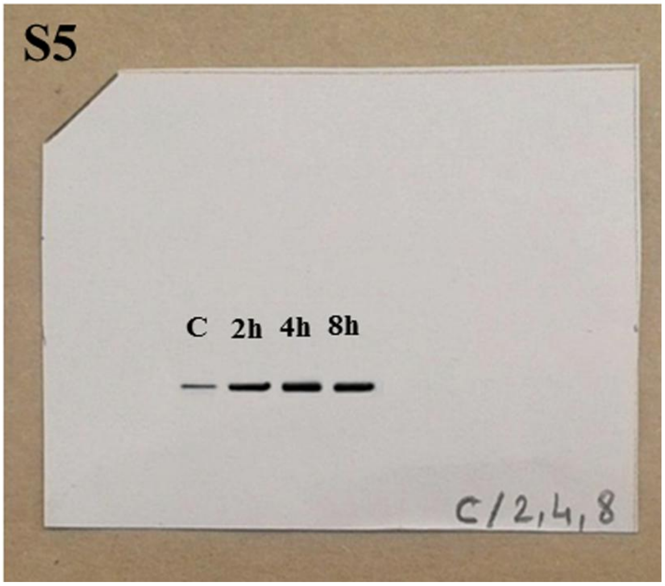

Figure S5: Representative full size image of western blotting membrane presented in the Figure 6C. Immunoblotting of PdPXG2 protein in control protoplasts and TCDD-treated protoplasts after exposure to 10 ng L^{-1} TCDD for 2, 4 and 8 h.

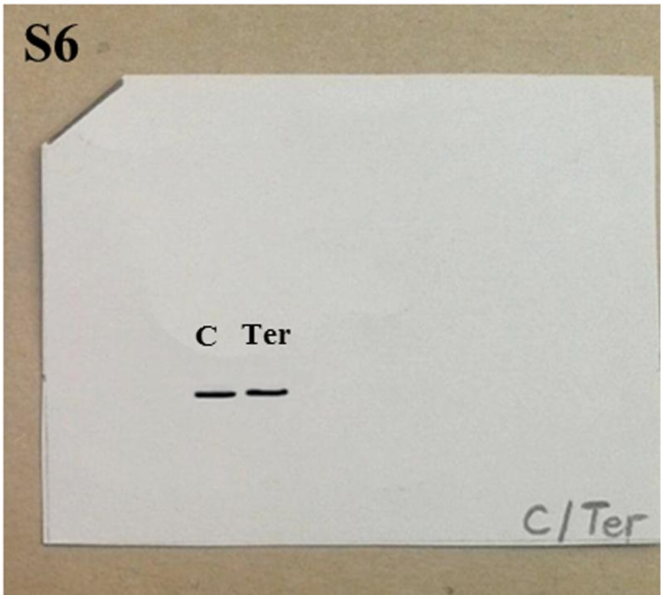

Figure S6: Representative full size image of western blotting membrane presented in the Figure 7B. Immunoblotting of PdPXG2 protein in control protoplasts and in terbufos-pretreated protoplasts.
